# Supplementary material for: Transition metal catalysis in the mitochondria of living cells
Source: Nat Commun. 2016 Sep 7;7:12538. doi: 10.1038/ncomms12538 (PMC5023949; doi:10.1038/ncomms12538)
Supplement: Supplementary Information — Supplementary Figures 1-25, Supplementary Tables 1-2, Supplementary Methods and Supplementary References [file ncomms12538-s1.pdf]

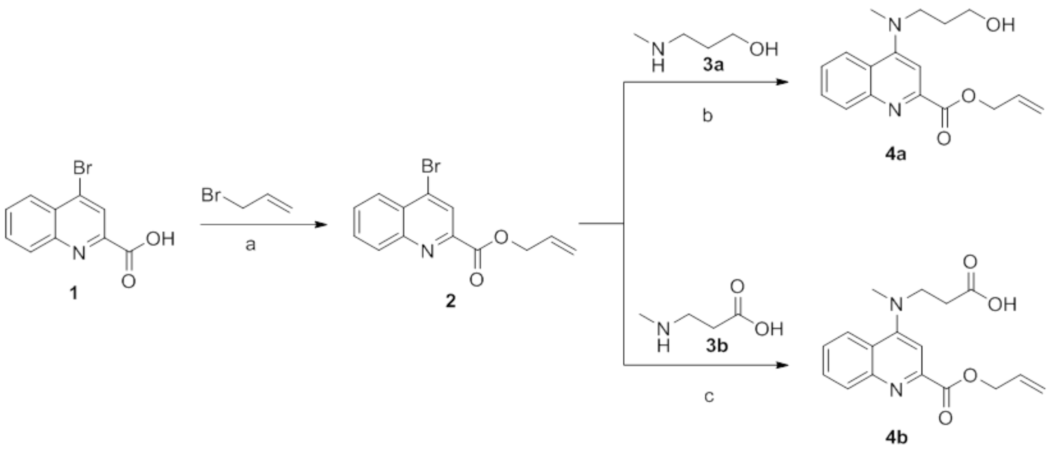

Supplementary Figure 1. Reagents: a) **1** (1.0 eq), allyl bromide (1.6 eq), $KHCO_3$ (1.7 eq), DMF, 12 h, 50 °C, 75%; b) **2** (1.0 q), **3a** (5.0 eq), DMSO, 1 h, 100 °C, 87%; c) **2** (1.0 q), **3b** (5.0 eq), DMSO, 1 h, 100 °C, 50%.

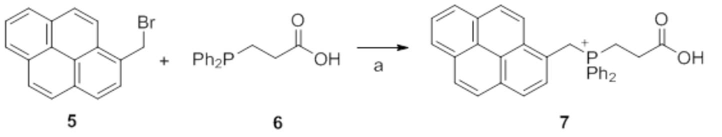

Supplementary Figure 2. Reagents: a) **5** (1.0 eq), **6** (1.5 eq), toluene, 12 h, 110 °C, 70%.

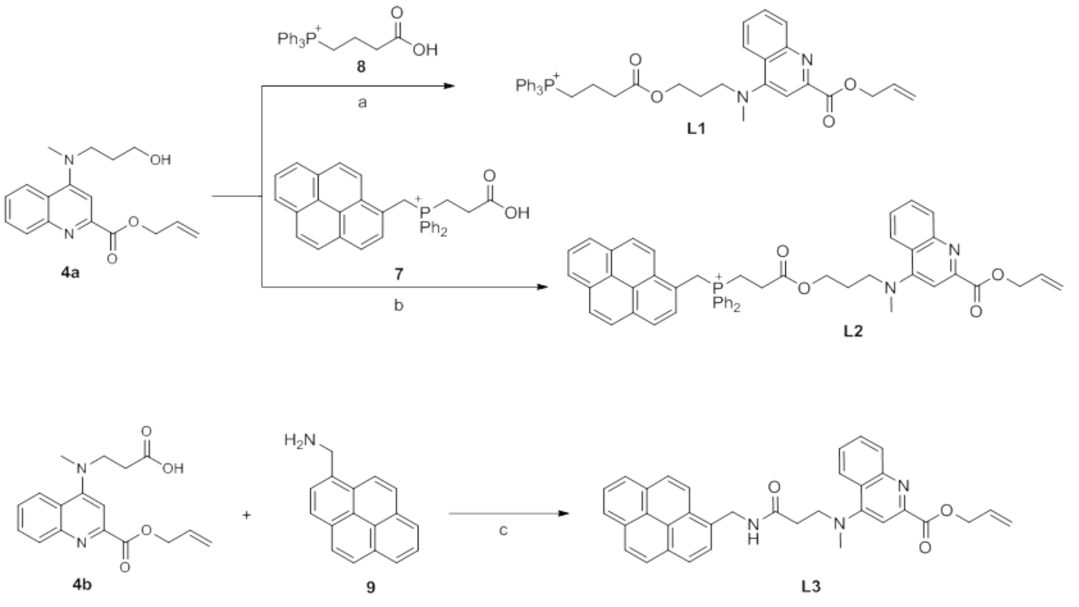

Supplementary Figure 3. Synthesis of the ligands **L1-L3**. Reagents and abbreviations: a) **4a** (1.0 eq), **8** (1.0 eq), EDC (2.5 eq), DMAP (2.0 eq), 12 h, room temperature, 30%; b) **4a** (1.0 eq), **7** (1.0 eq), EDC (2.5 eq), DMAP (2.0 eq), 12 h, room temperature, 26%; c) **4b** (1.0 eq), **9** (1.0 eq), EDC (2.5 eq), DMAP (2.0

eq), 12 h, room temperature, 55%. EDC = 1-Ethyl-3-(3-dimethylaminopropyl)carbodiimide; DMAP = 4-Dimethylaminopyridine.

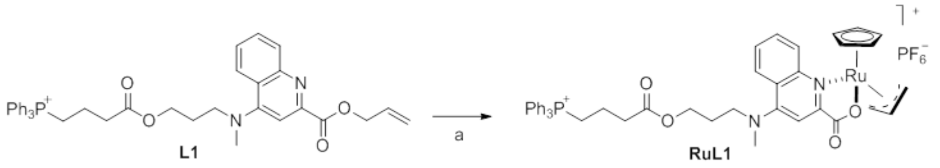

Supplementary Figure 4. Reagents: a) **L1** (1.0 eq), $[RuCp(CH_3CN)_3]PF_6$ (1.0 eq), DCM, 12 h, room temperature, 65%.

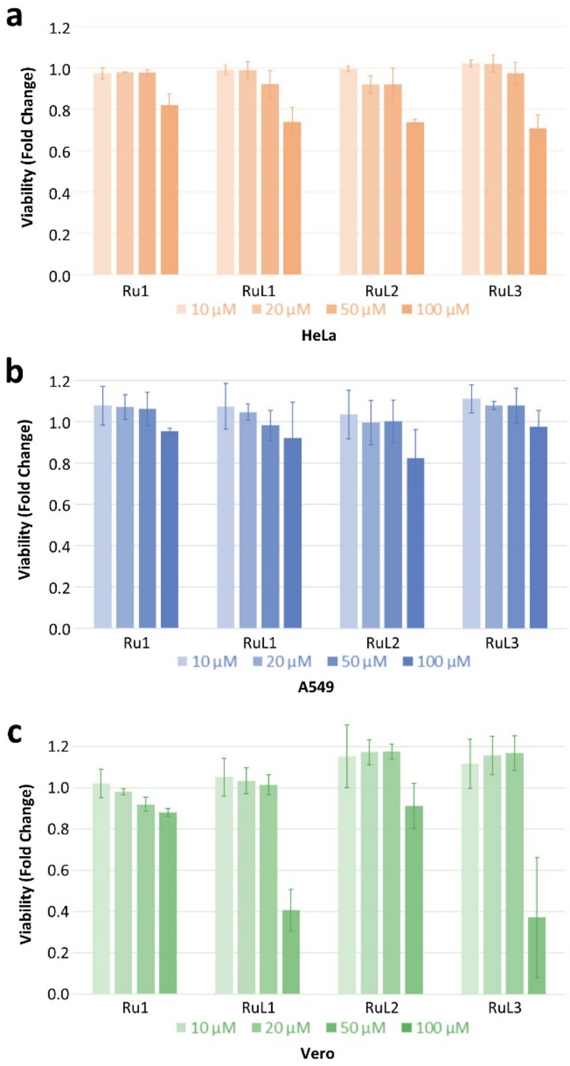

Supplementary Figure 5. Viability Assays. a, HeLa, **b**, A549 and **c**, Vero cells were incubated in cell culture medium containing the indicated amounts of the catalysts **Ru1**, **RuL1**-**RuL3** for 24 h and the amount of viable cells was analysed by MTT assay. The viability is expressed as the fold change of the

absorbance value with respect to untreated cells (value 1.0). We could observe a decrease in the viability of cells (of around 30%) only at concentrations of 100 μM.

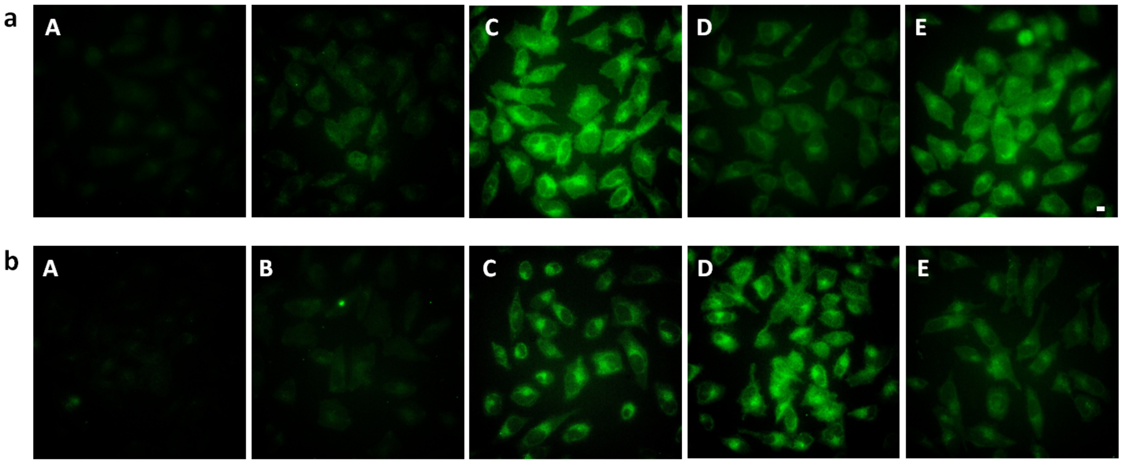

Supplementary Figure 6. Ru1, RuL1-RuL3-catalyzed uncaging of Rho-alloc in HeLa cells after PBS washings and observed under the microscope. a, **Rho-alloc** preincubation, **Rho** fluorescence after addition of A) No catalyst, B) **Ru1**, C) **RuL1**, D) **RuL2** and E) **RuL3**. **b**, Catalyst preincubation, **Rho** fluorescence in cells pretreated with A) No catalyst, B) **Ru1**, C) **RuL1**, D) **RuL2** and E) **RuL3**. Scale bar: 12.5 μm.

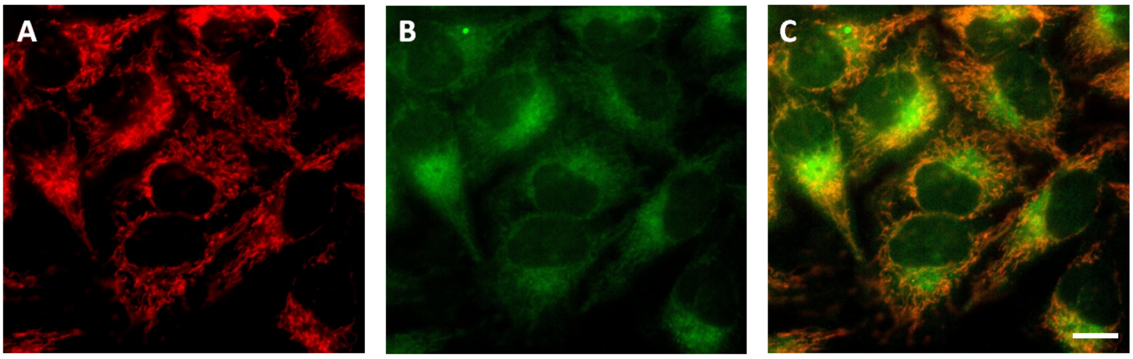

Supplementary Figure 7. RuL1-catalyzed uncaging of Rho-alloc in HeLa cells and observed under the microscope. A) Mitochondrial labeling with TMRE (red), B) **Rho** fluorescence in cells pretreated with **Ru1** and C) merging of A and B. Scale bar: 12.5 μm.

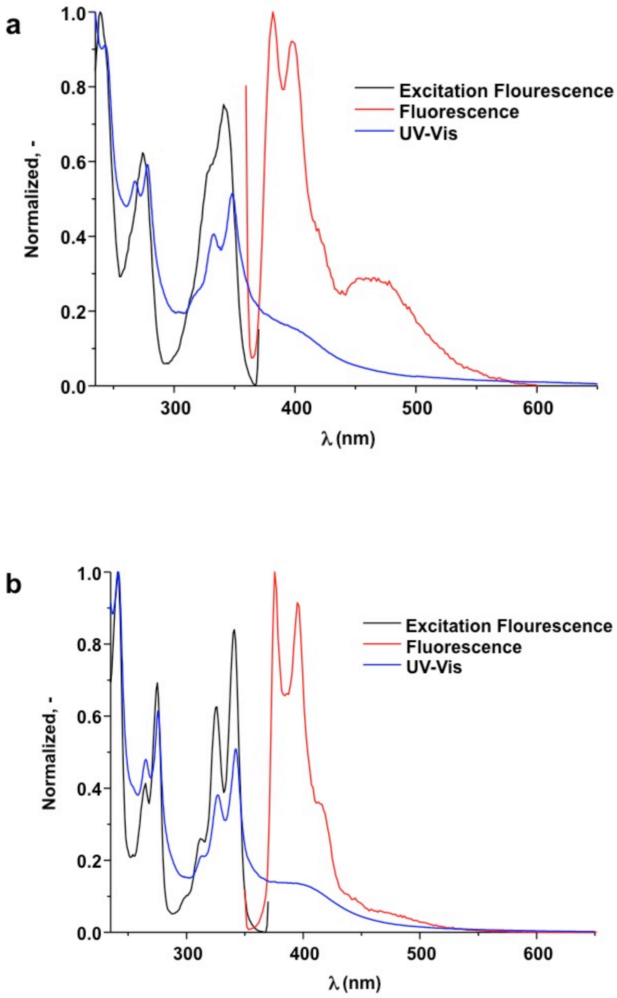

Supplementary Figure 8. UV-vis, fluorescence and emission excitation spectra of **RuL2 (a)** and **RuL3 (b)**.

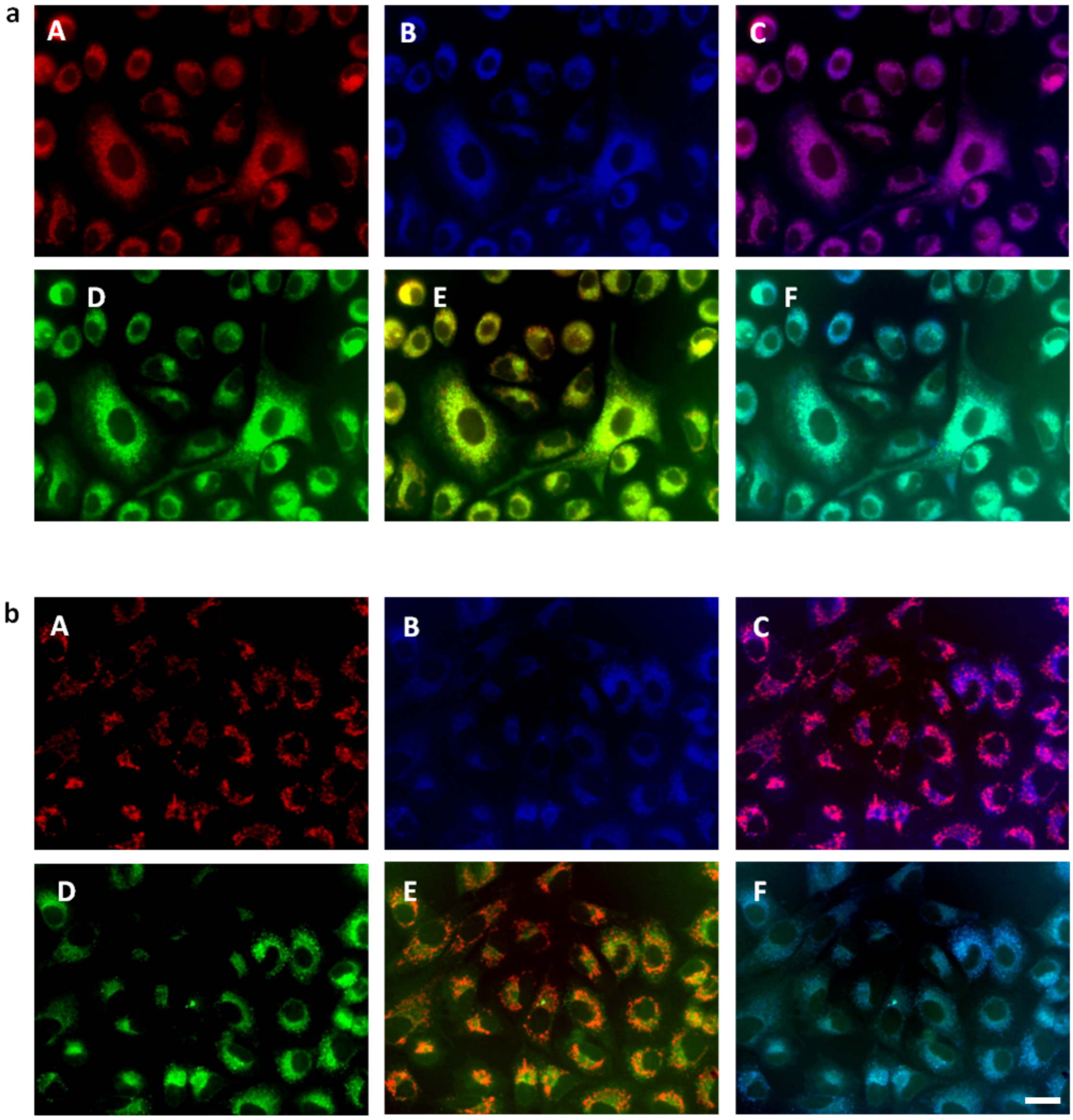

Supplementary Figure 9. Reactivity of Ruthenium complexes RuL2 and RuL3 in A549 cells. **a**, Metal complex **RuL2** and **b**, **RuL3**. A) Mitochondrial labeling with TMRE (red), B) emission of the metal complex (blue), C) merging of A and B, D) **Rho** fluorescence in cells pretreated with the ruthenium catalysts, E) merging of A and D and F) merging of B and D. Scale bar: 12.5 µm.

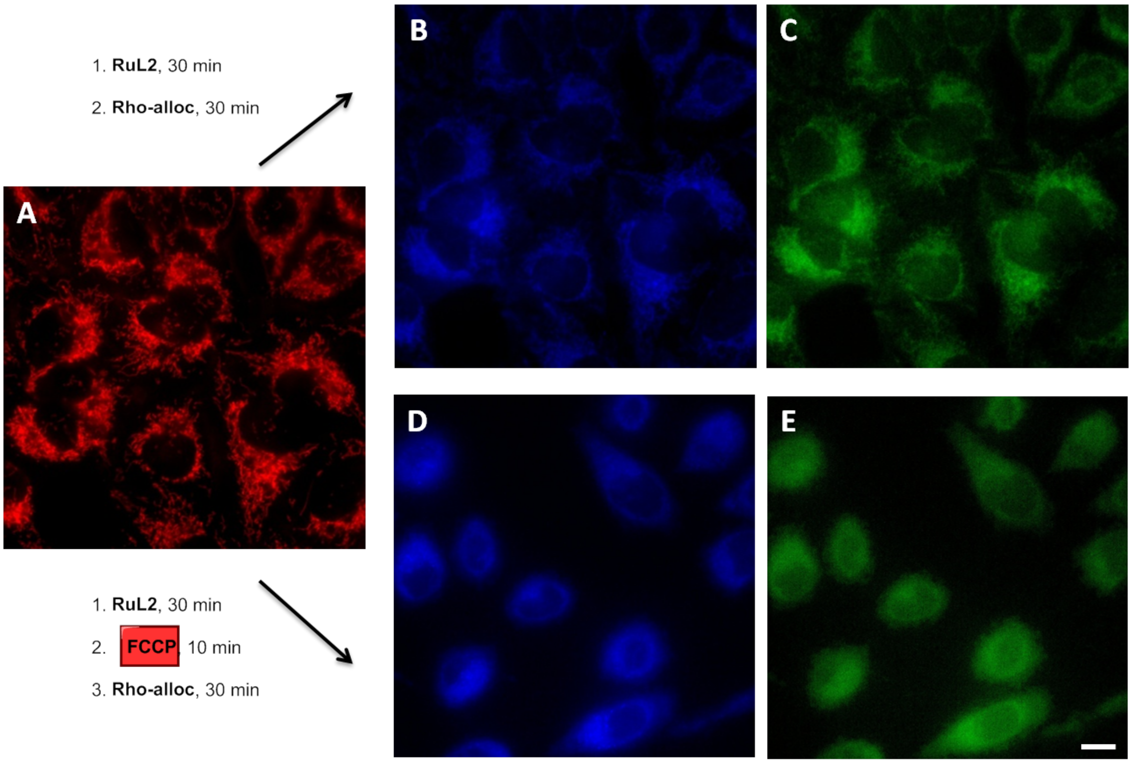

Supplementary Figure 10. Depolarization of mitochondrial membrane of HeLa cells. A) Mitochondrial labeling with MitoTracker red (200 nM), B) emission of **RuL2** (blue) in absence of FCCP, C) **Rho** fluorescence in cells pretreated with **RuL2** in absence of FCCP, D) emission of **RuL2** (blue) in cells treated with FCCP, E) **Rho** fluorescence in cells pretreated with **RuL2** and FCCP. Scale bar: 12.5 µm.

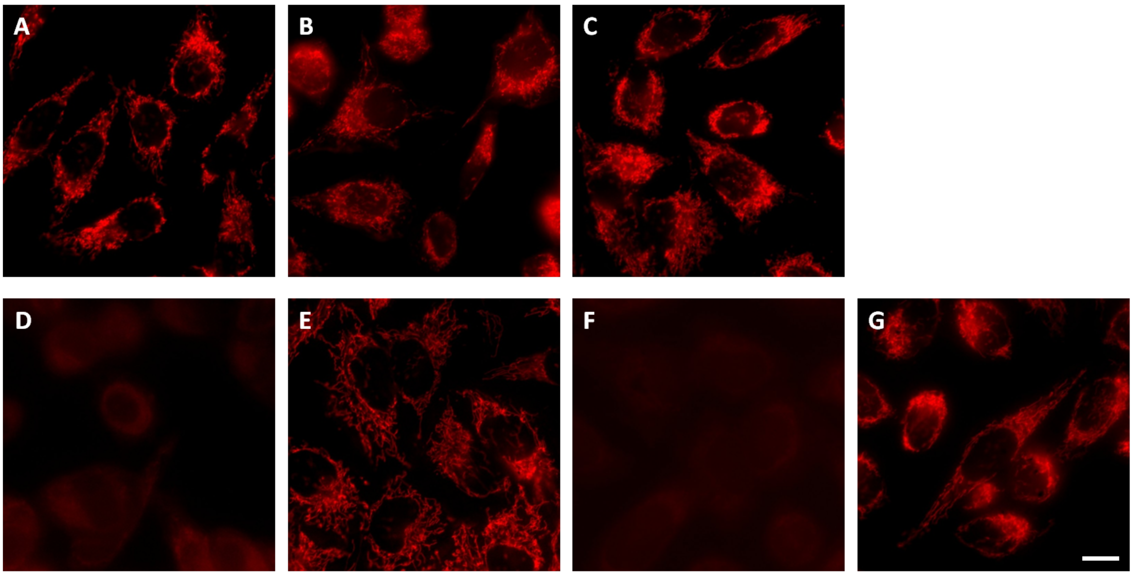

Supplementary Figure 11. Activation of DNP-allyl inside HeLa cells. Fluorescence micrographs of the mitochondrial staining with TMRE (100 nM) of A) untreated control cells, B) cells incubated with **RuL2** (50 µM), C) cells incubated with **RuL3** (50 µM), D) cells treated with **DNP** (500 µM), E) cells treated with **DNP-allyl** (150 µM), F) cells treated with **DNP-allyl** (150 µM) after incubation with **RuL2** and G) cells treated with **DNP-allyl** (150 µM) after incubation with**RuL3**. Scale bar: 12.5 µm.

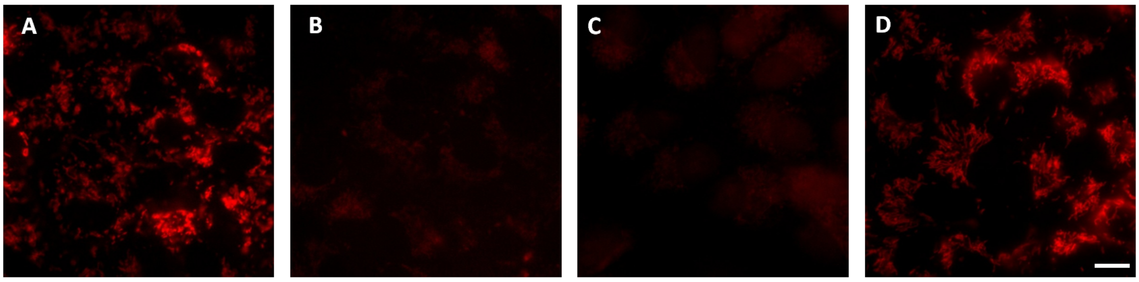

Supplementary Figure 12. Activation of DNP-allyl inside Vero cells. A) Cells treated with **DNP-allyl** (150 µM), B) cells treated with **DNP** (500 µM), C) cells treated with **DNP-allyl** (150 µM) after incubation with **RuL2** and D) cells treated with **DNP-allyl** (150 µM) after incubation with **RuL3**. Scale bar: 12.5 µm.

a

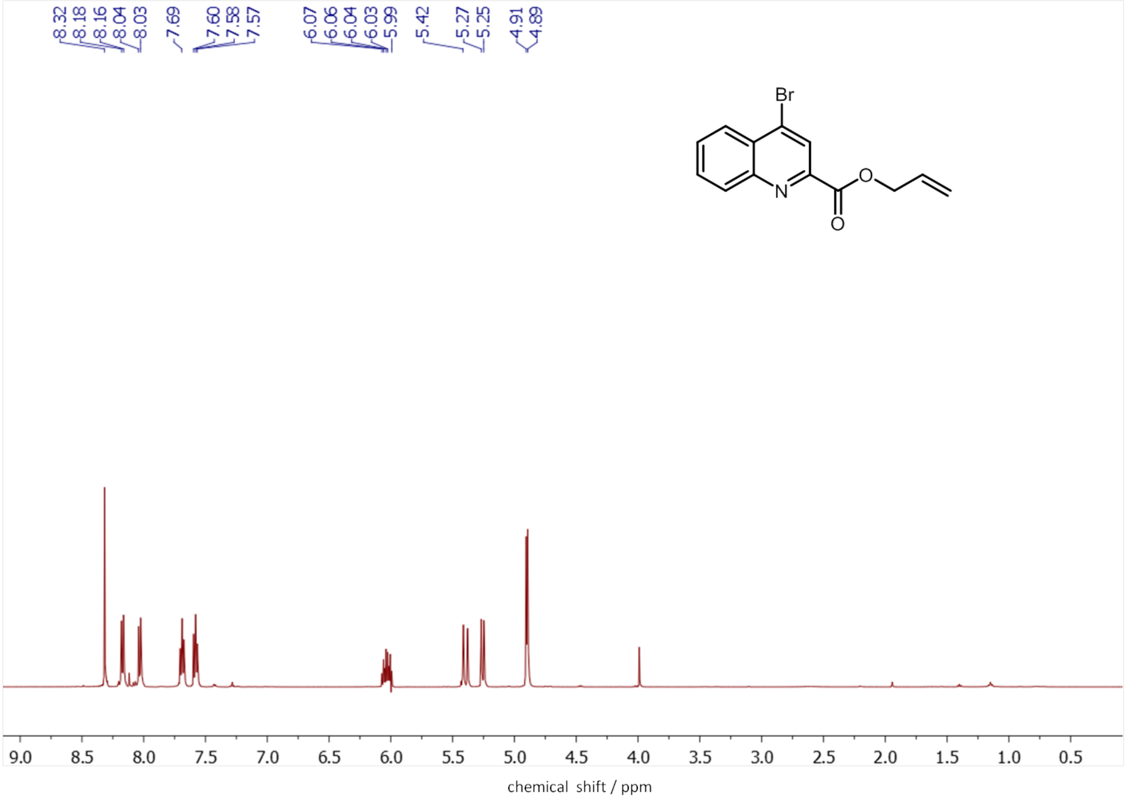

b

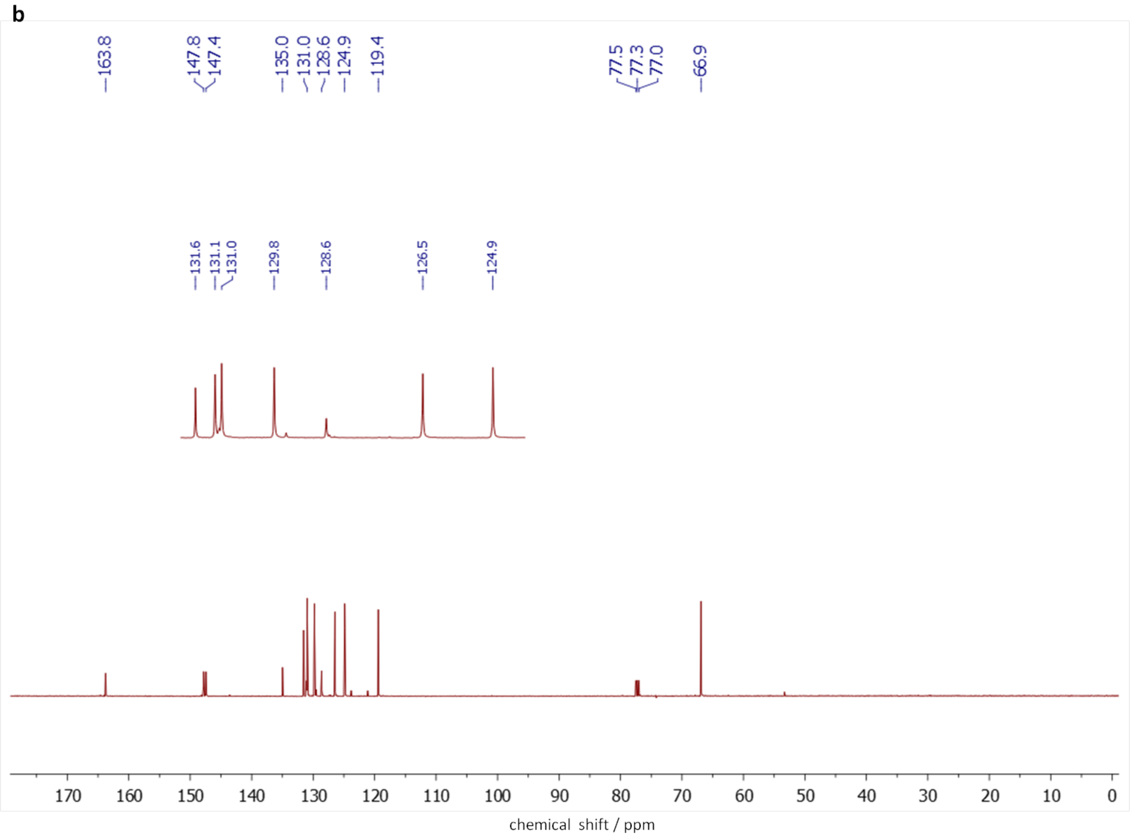

c

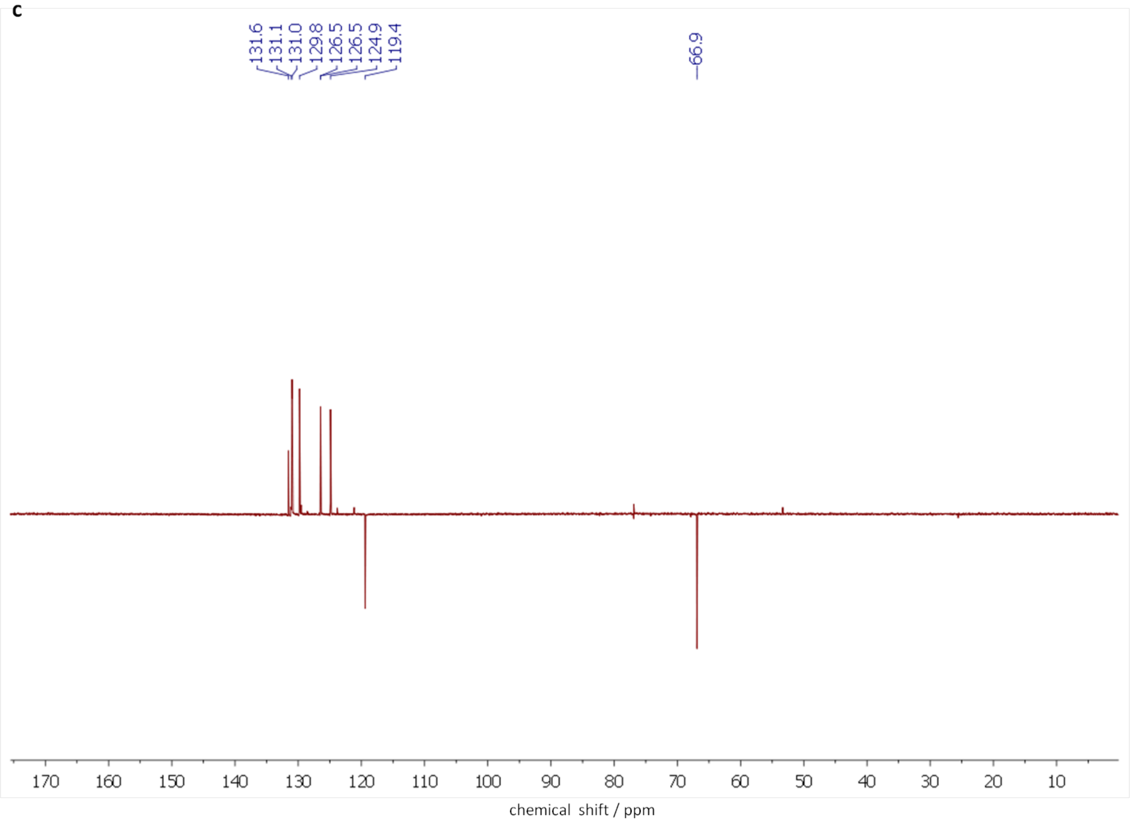

Supplementary Figure 13. a, ^{1}H NMR, **b**, ^{13}C NMR and **c**, DEPT NMR of **2**.

a

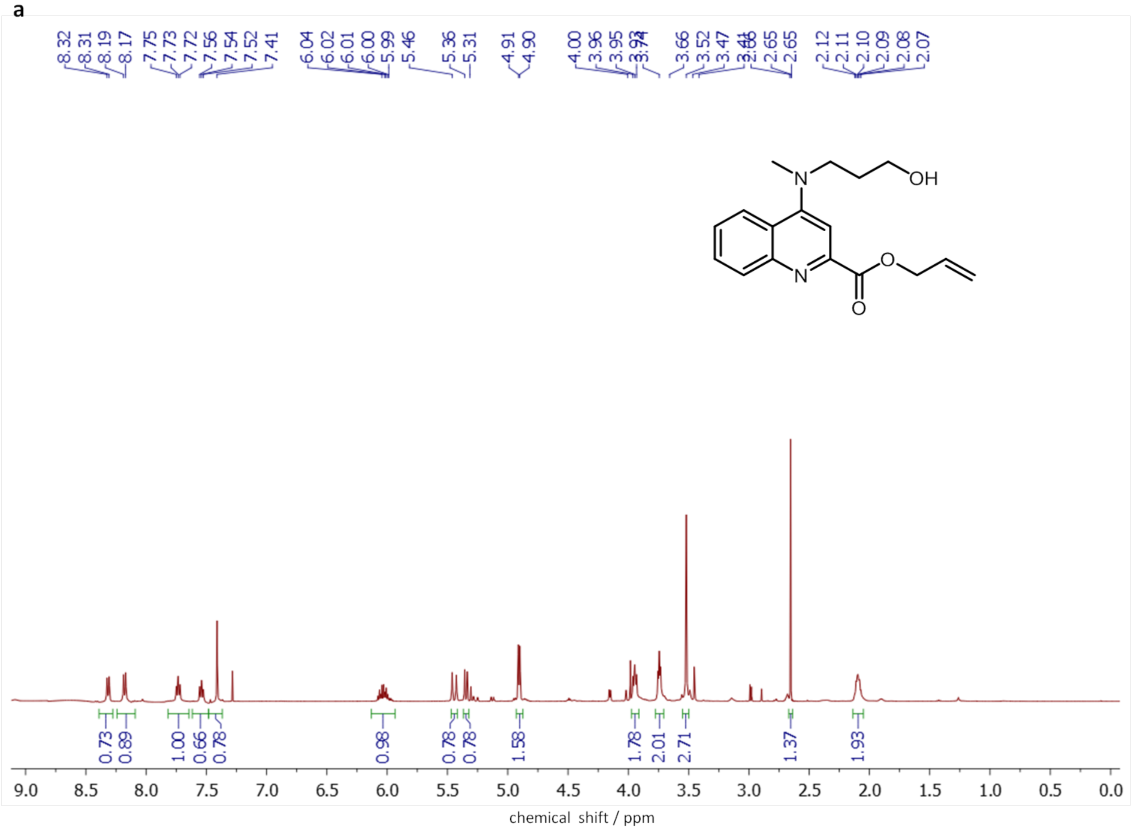

8.32 8.31 8.19 8.17 7.75 7.73 7.72 7.56 7.54 7.52 7.41 6.04 6.02 6.01 6.00 5.99 5.46 5.36 5.31 4.91 4.90 4.00 3.96 3.95 3.93 3.74 3.66 3.52 3.47 3.41 3.46 2.65 2.65 2.12 2.11 2.10 2.09 2.08 2.07
N
OH
N
O
O
0.73 0.89 1.00 0.66 0.78 0.98 0.78 0.78 1.58 1.78 2.01 2.71 1.37 1.93
9.0 8.5 8.0 7.5 7.0 6.5 6.0 5.5 5.0 4.5 4.0 3.5 3.0 2.5 2.0 1.5 1.0 0.5 0.0
chemical shift / ppm

b

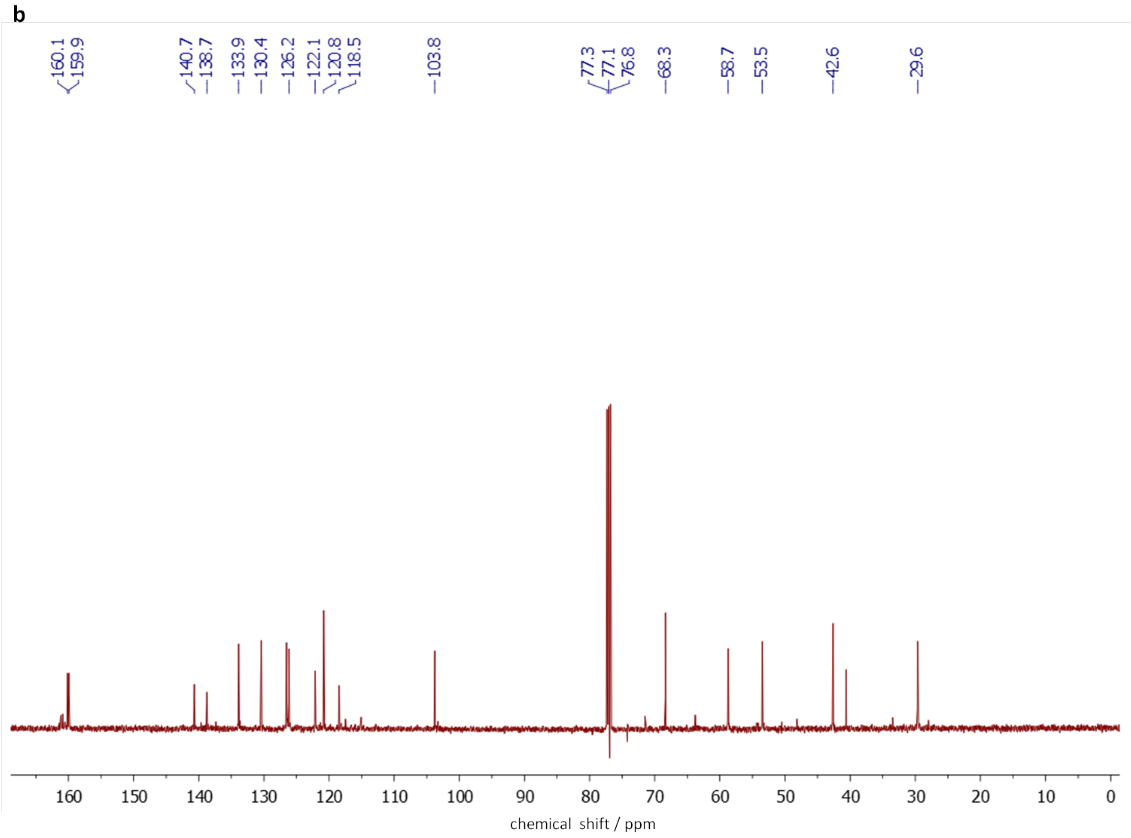

160.1 159.9 140.7 138.7 133.9 130.4 126.2 122.1 120.8 118.5 103.8 77.3 77.1 76.8 68.3 58.7 53.5 42.6 29.6
160 150 140 130 120 110 100 90 80 70 60 50 40 30 20 10 0
chemical shift / ppm

c

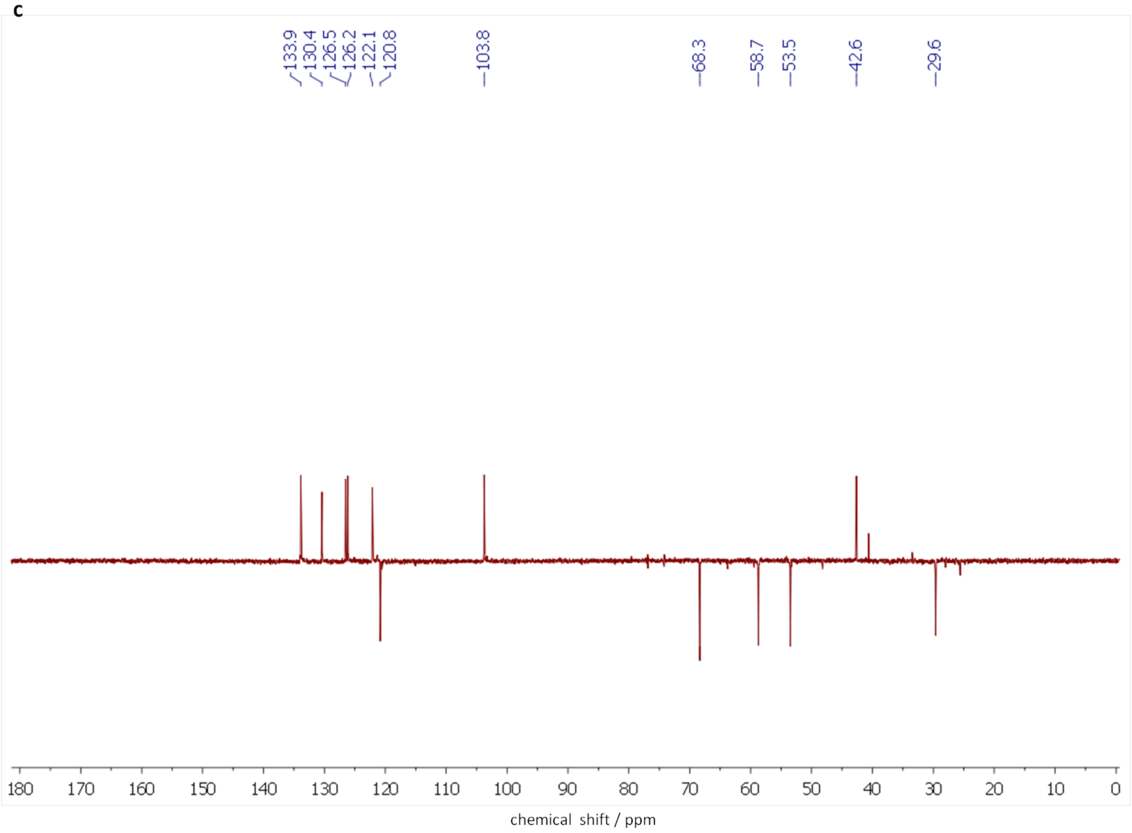

Supplementary Figure 14. a, ^{1}H NMR, **b**, ^{13}C NMR and **c**, DEPT NMR of **4a**.

a

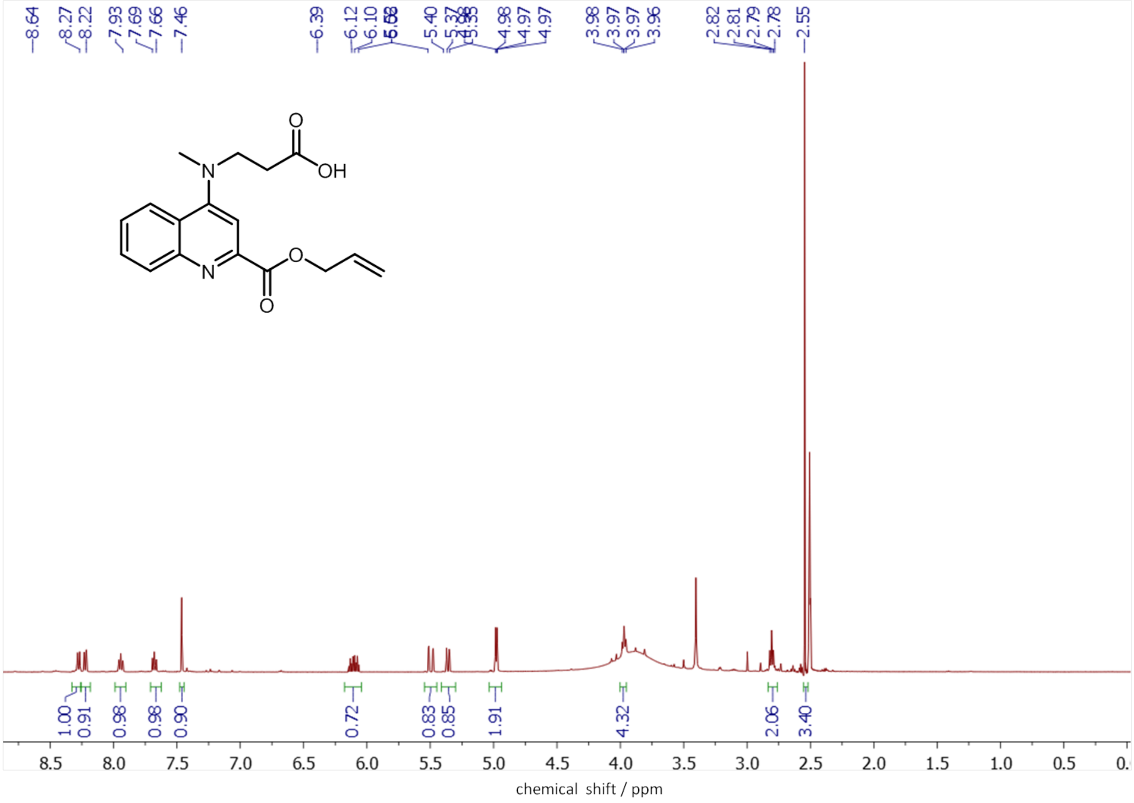

b

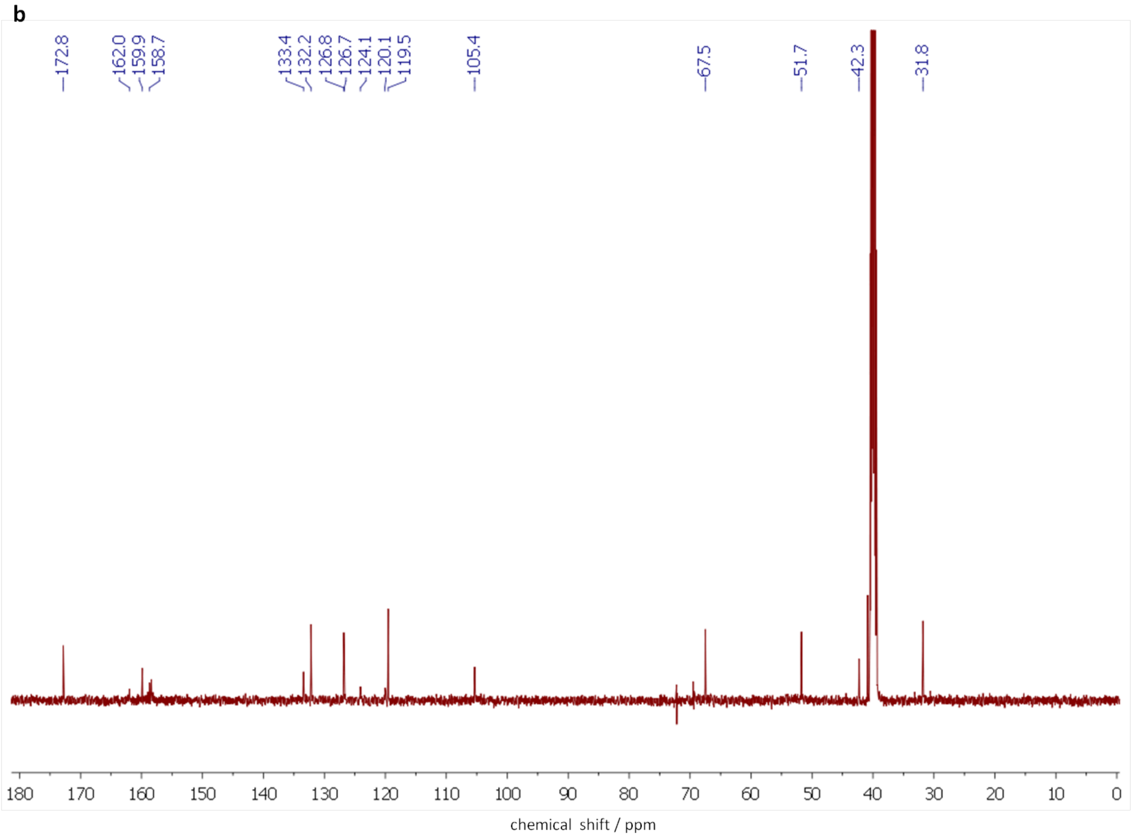

c

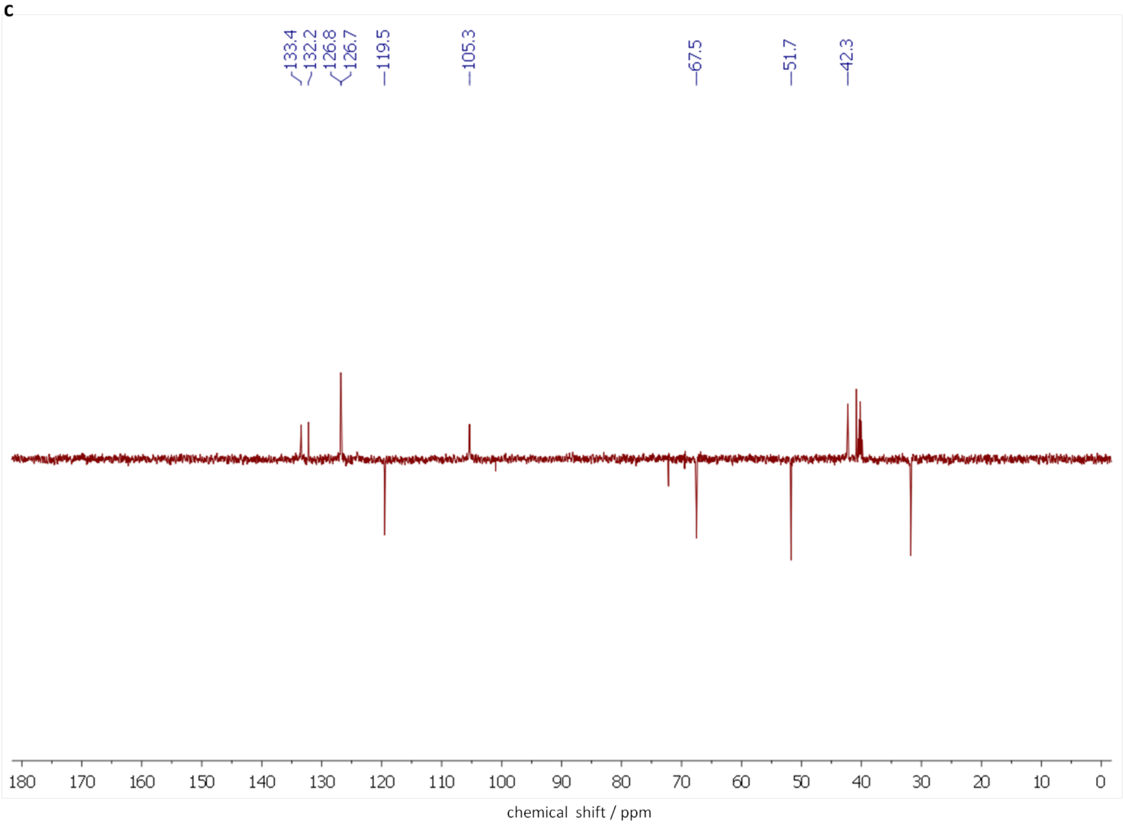

Supplementary Figure 15. **a**, ^{1}H NMR, **b**, ^{13}C NMR and **c**, DEPT NMR of **4b**.

a

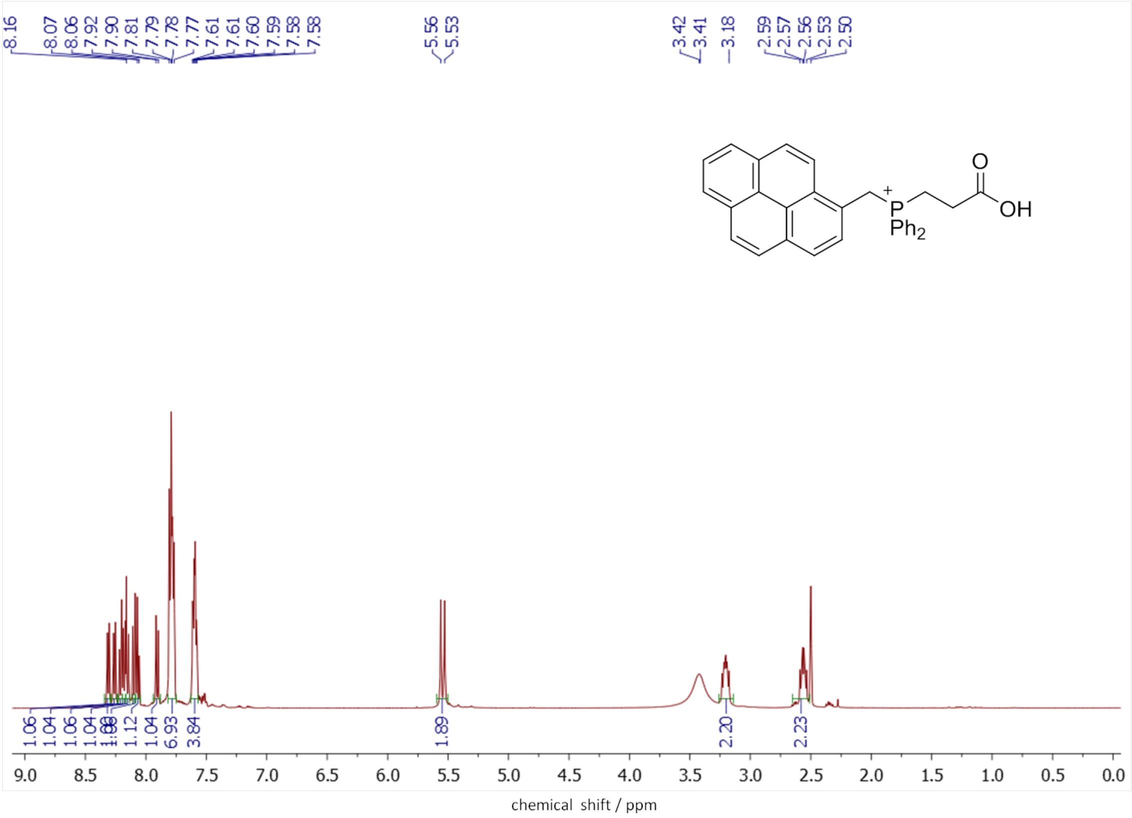
8.16
8.07 8.06 7.92 7.90 7.81 7.79 7.78 7.77 7.61 7.61 7.60 7.59 7.58 7.58
5.56 5.53
3.42 3.41 3.18
2.59 2.57 2.56 2.53 2.50
O
OH
P
Ph2
1.06 1.04 1.06 1.04 1.12 1.04 6.93 3.84
1.89
2.20
2.23
9.0 8.5 8.0 7.5 7.0 6.5 6.0 5.5 5.0 4.5 4.0 3.5 3.0 2.5 2.0 1.5 1.0 0.5 0.0
chemical shift / ppm

b

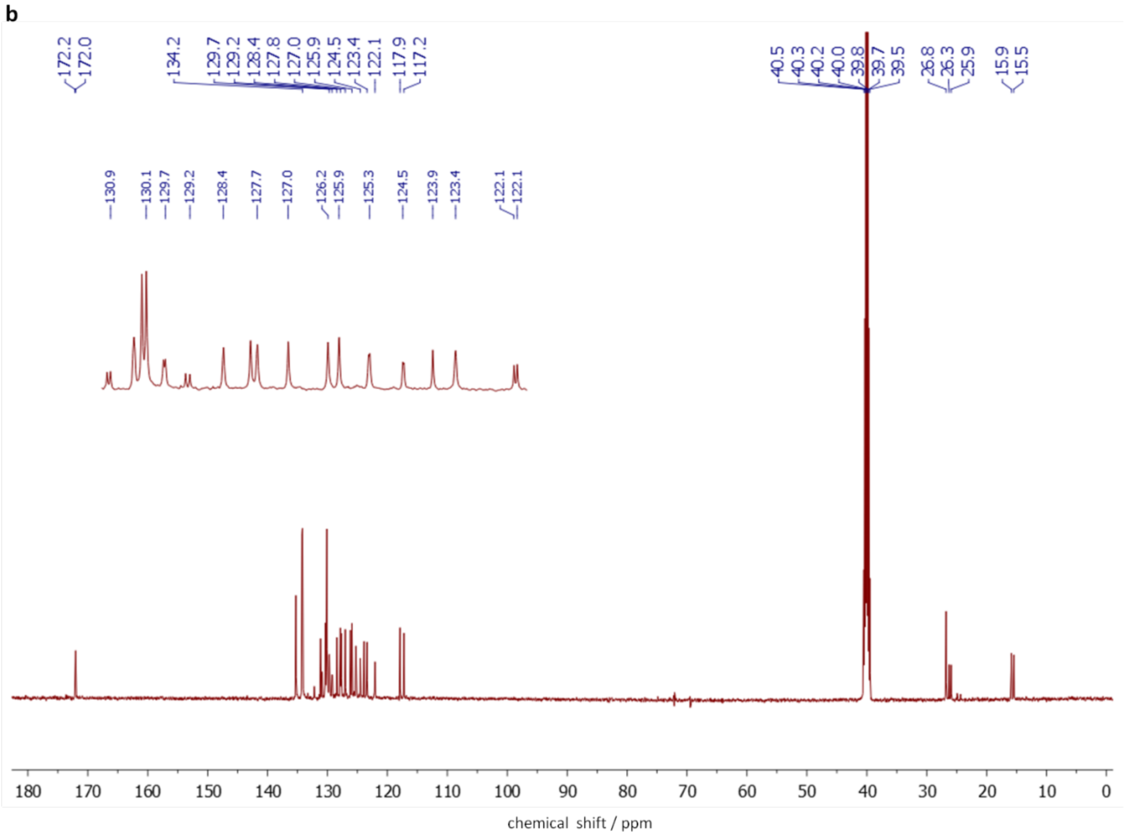
172.2 172.0
134.2
129.7 129.2 128.4 127.8 127.0 125.9 124.5 123.4 122.1
117.9 117.2
40.5 40.3 40.2 40.0 39.8 39.7 39.5
26.8 26.3 25.9
15.9 15.5
130.9 130.1 129.7 129.2 128.4 127.7 127.0 126.2 125.9 125.3 124.5 123.9 123.4 122.1 122.1
180 170 160 150 140 130 120 110 100 90 80 70 60 50 40 30 20 10 0
chemical shift / ppm

c

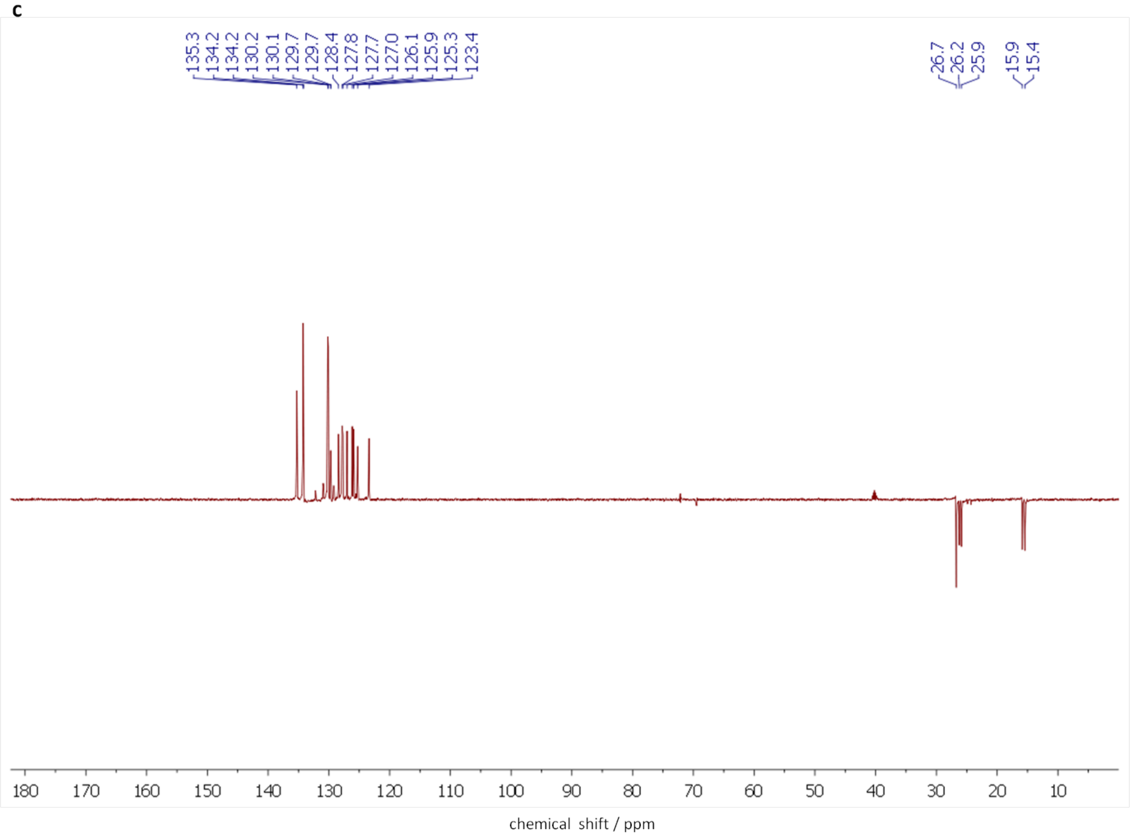

d

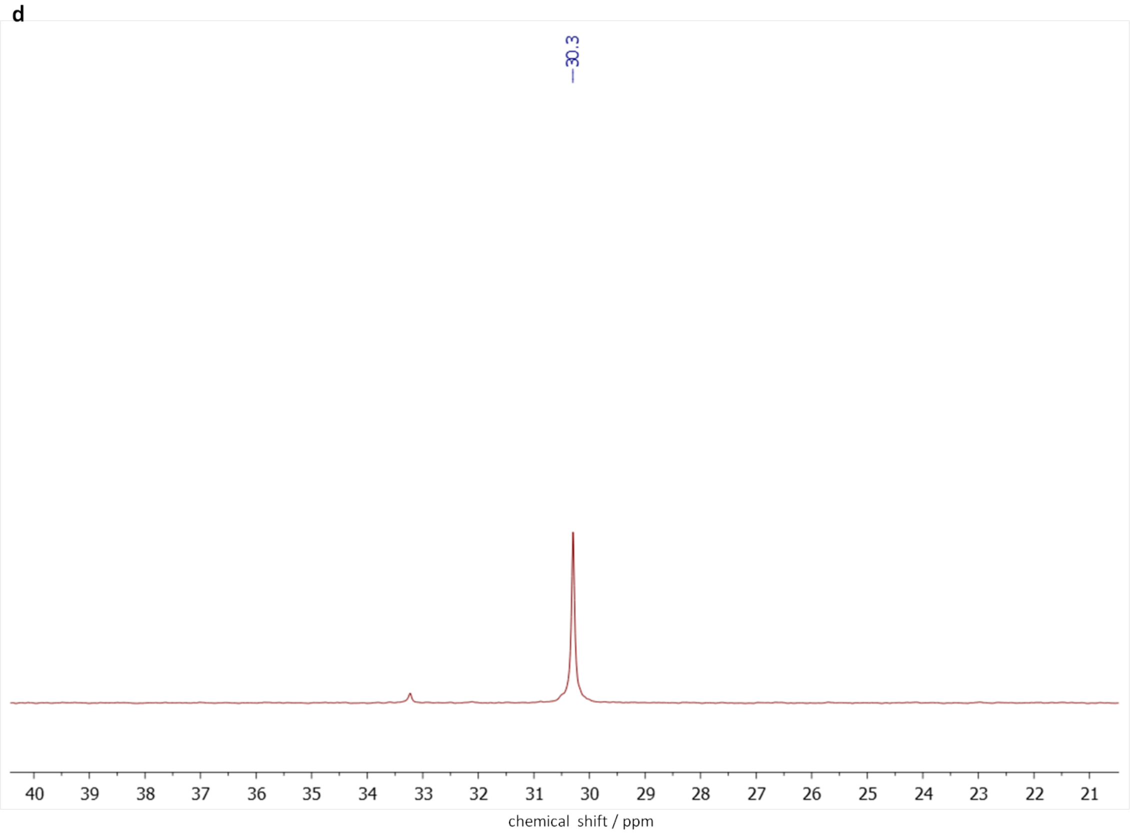

Supplementary Figure 16. **a**, ^{1}H NMR, **b**, ^{13}C NMR, **c**, DEPT NMR and **d**, ^{31}P of **7**.

a

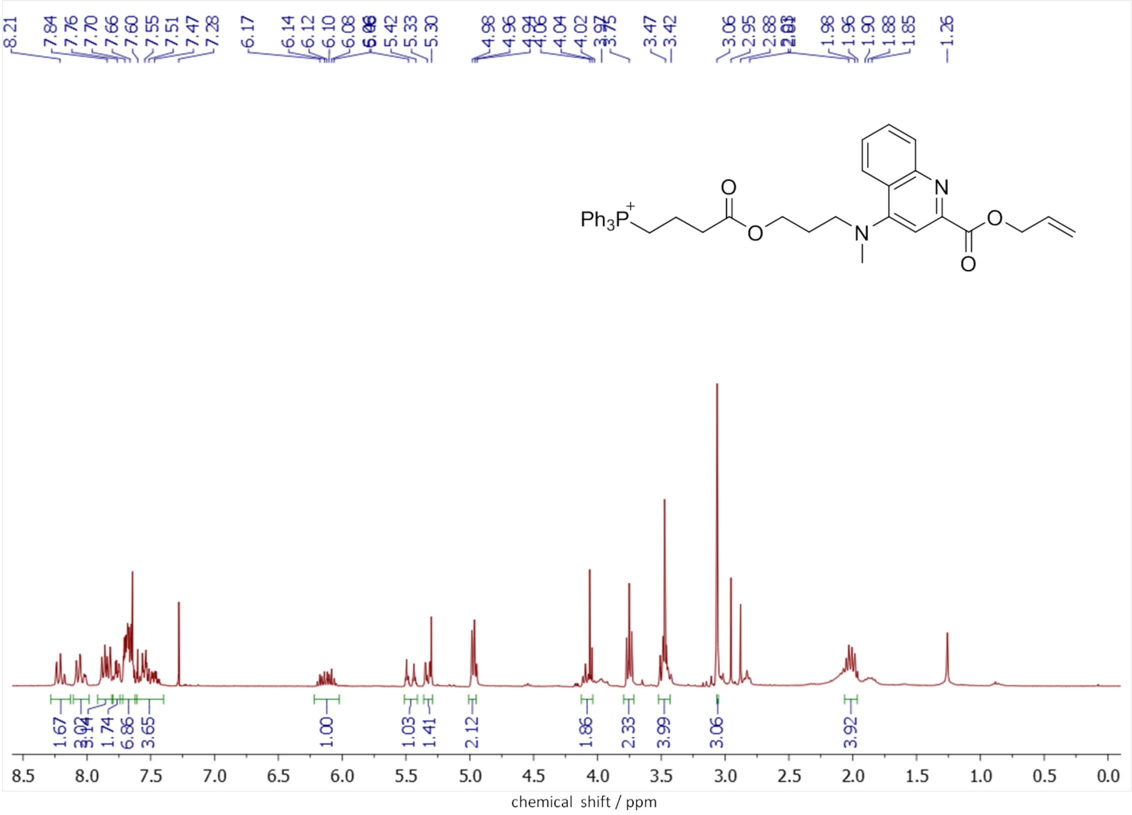

b

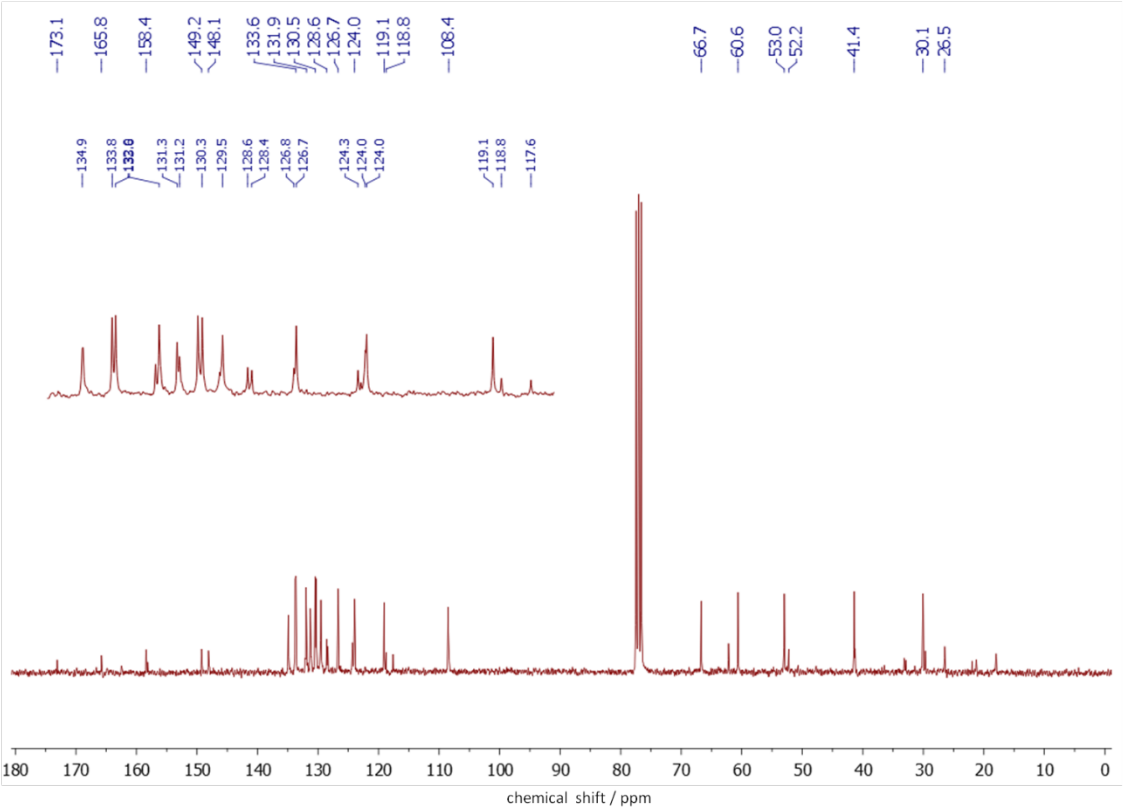

c

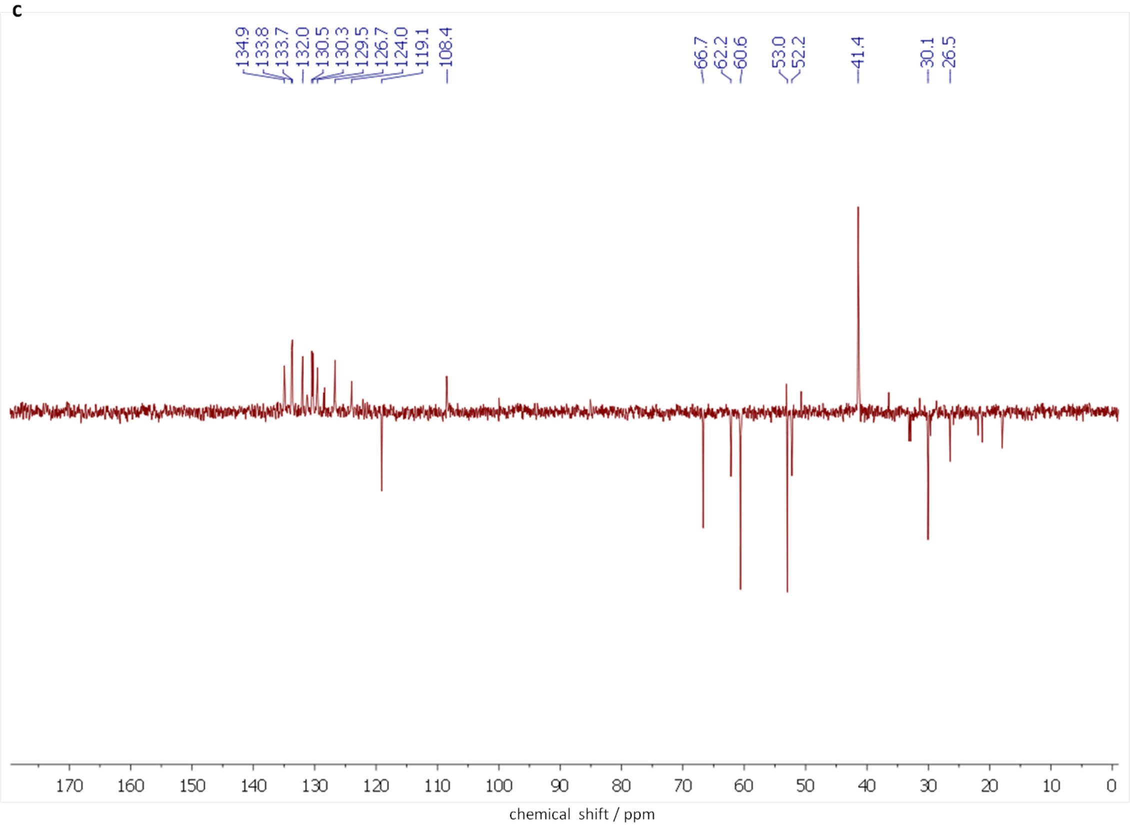

d

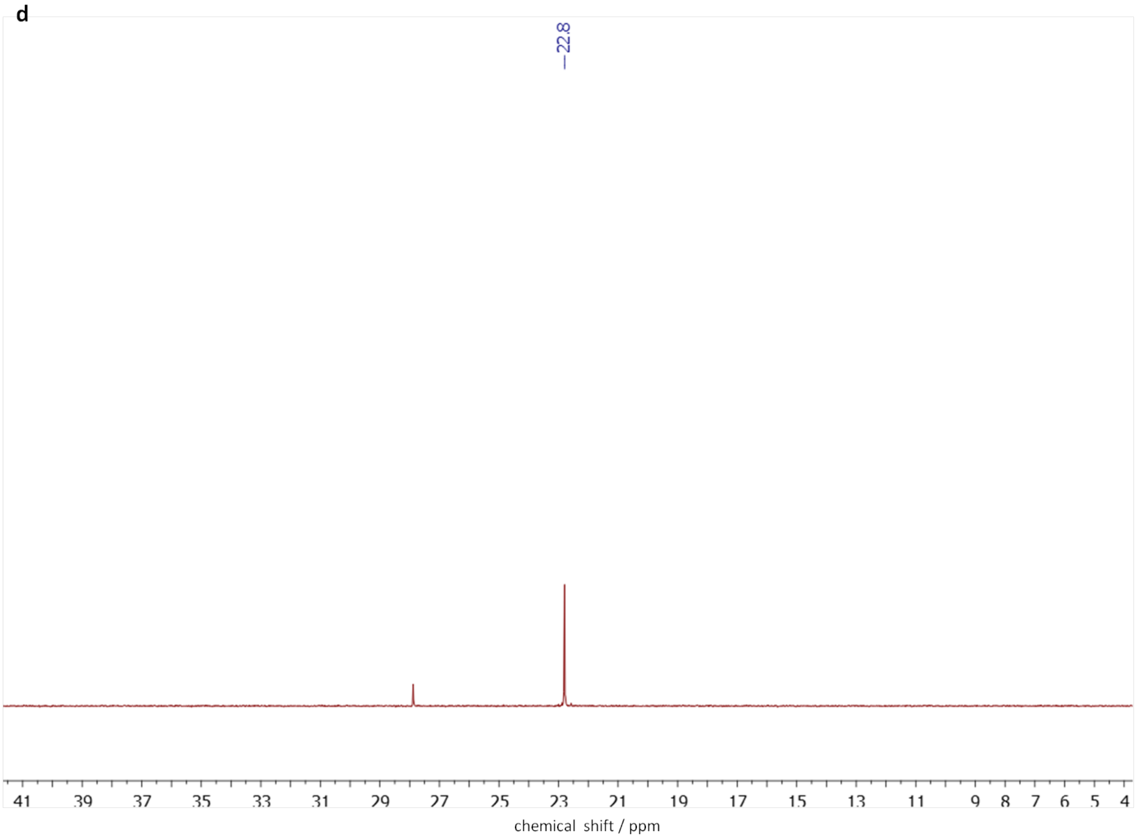

Supplementary Figure 17. **a**, ^{1}H NMR, **b**, ^{13}C NMR, **c**, DEPT NMR and **d**, ^{31}P of **L1**.

a

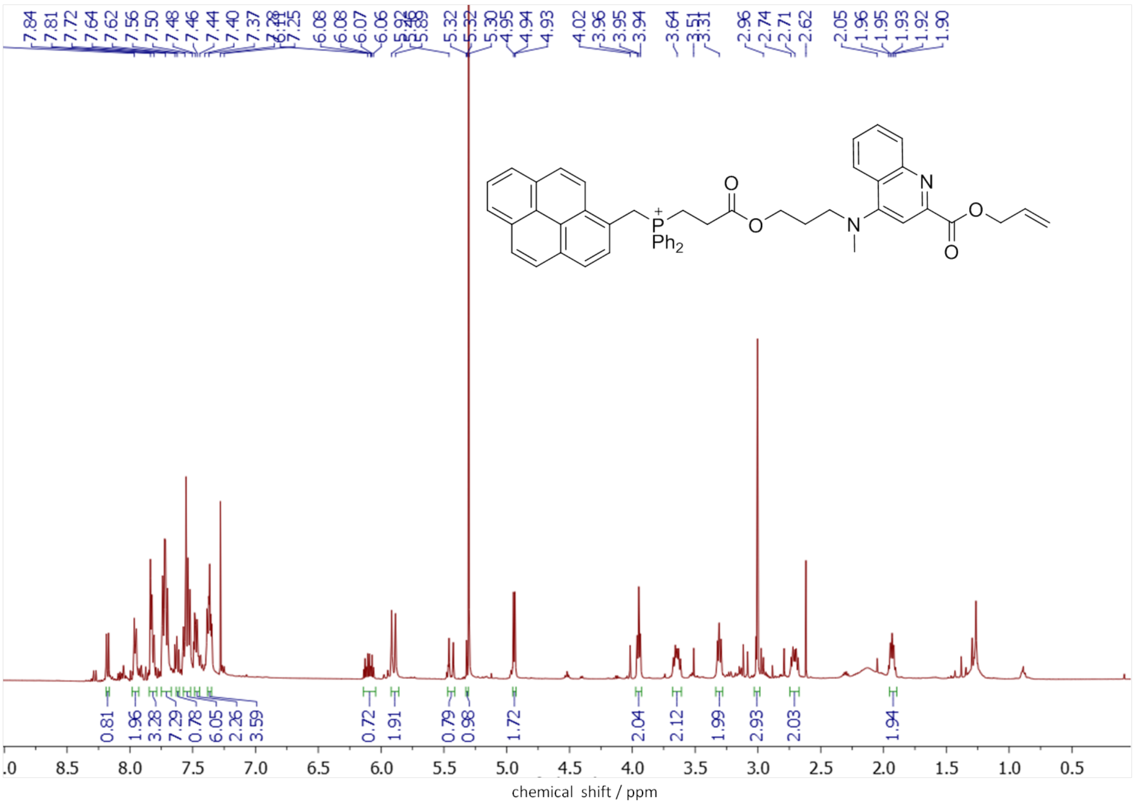

b

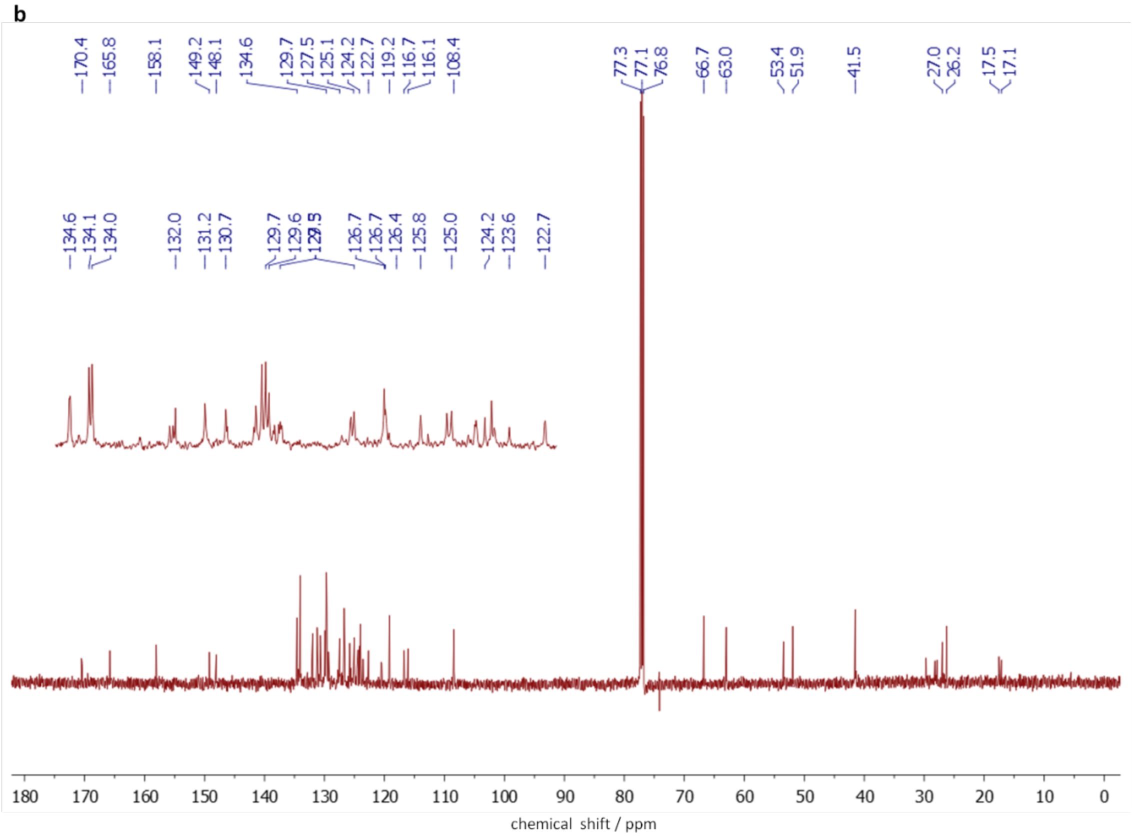

c

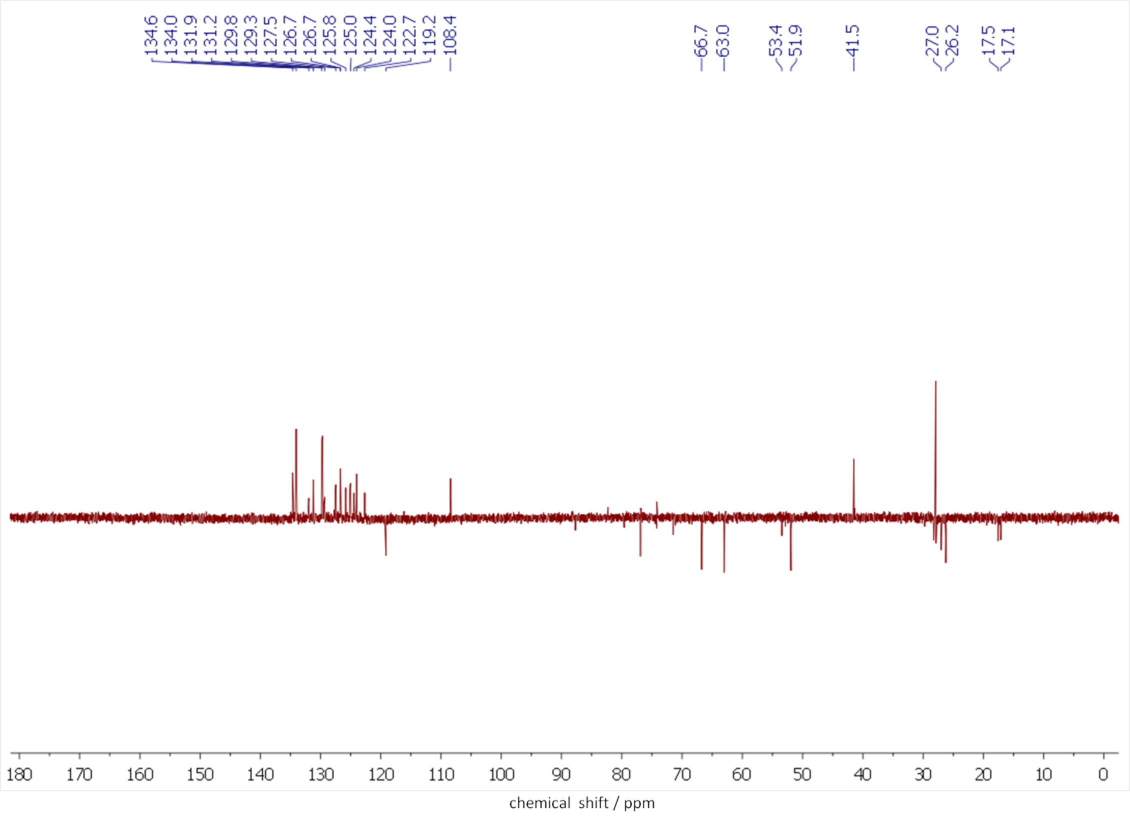

d

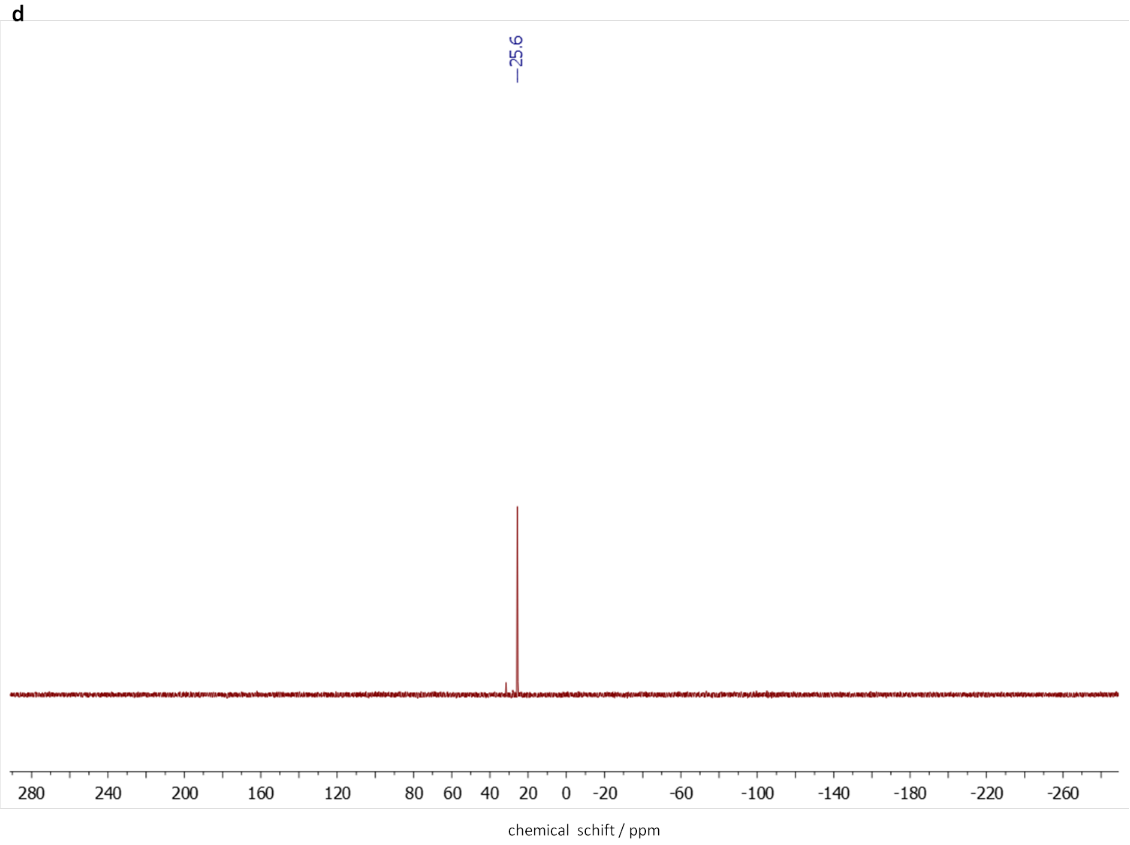

Supplementary Figure 18. **a**, ^{1}H NMR, **b**, ^{13}C NMR ,**c**, DEPT NMR and d) ^{31}P of **L2**.

a

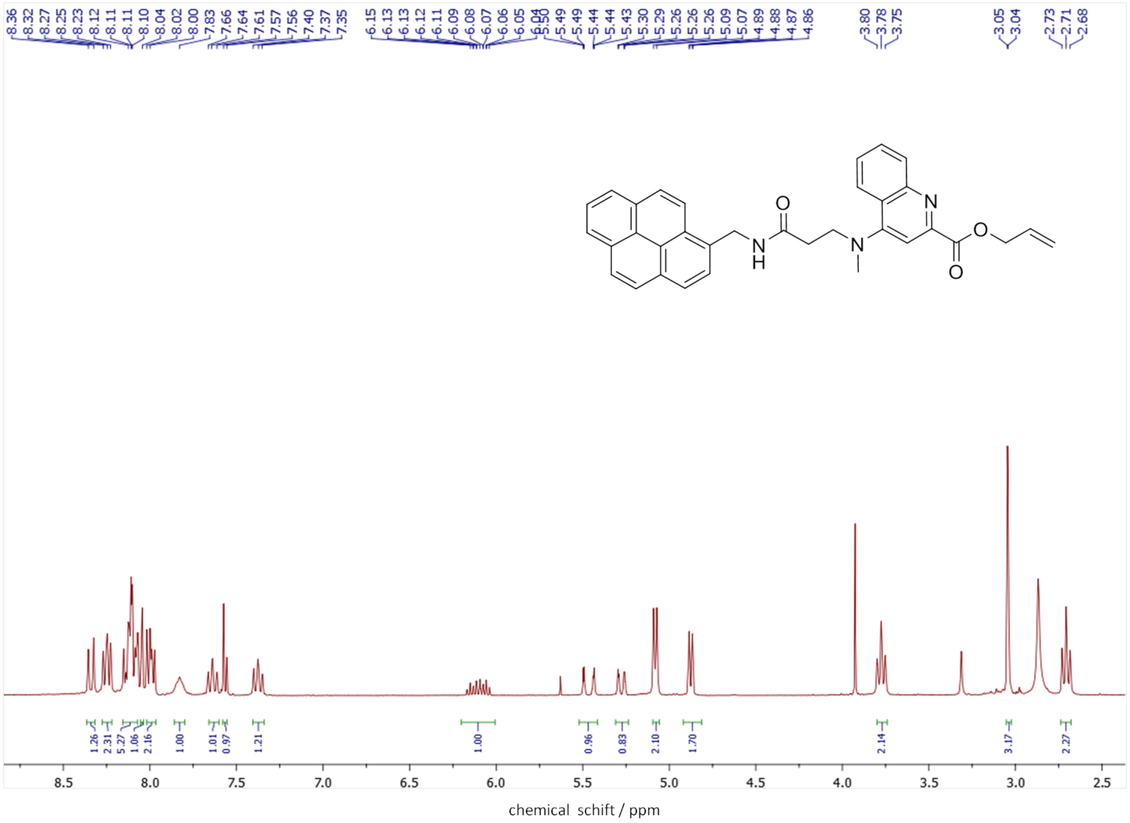

b

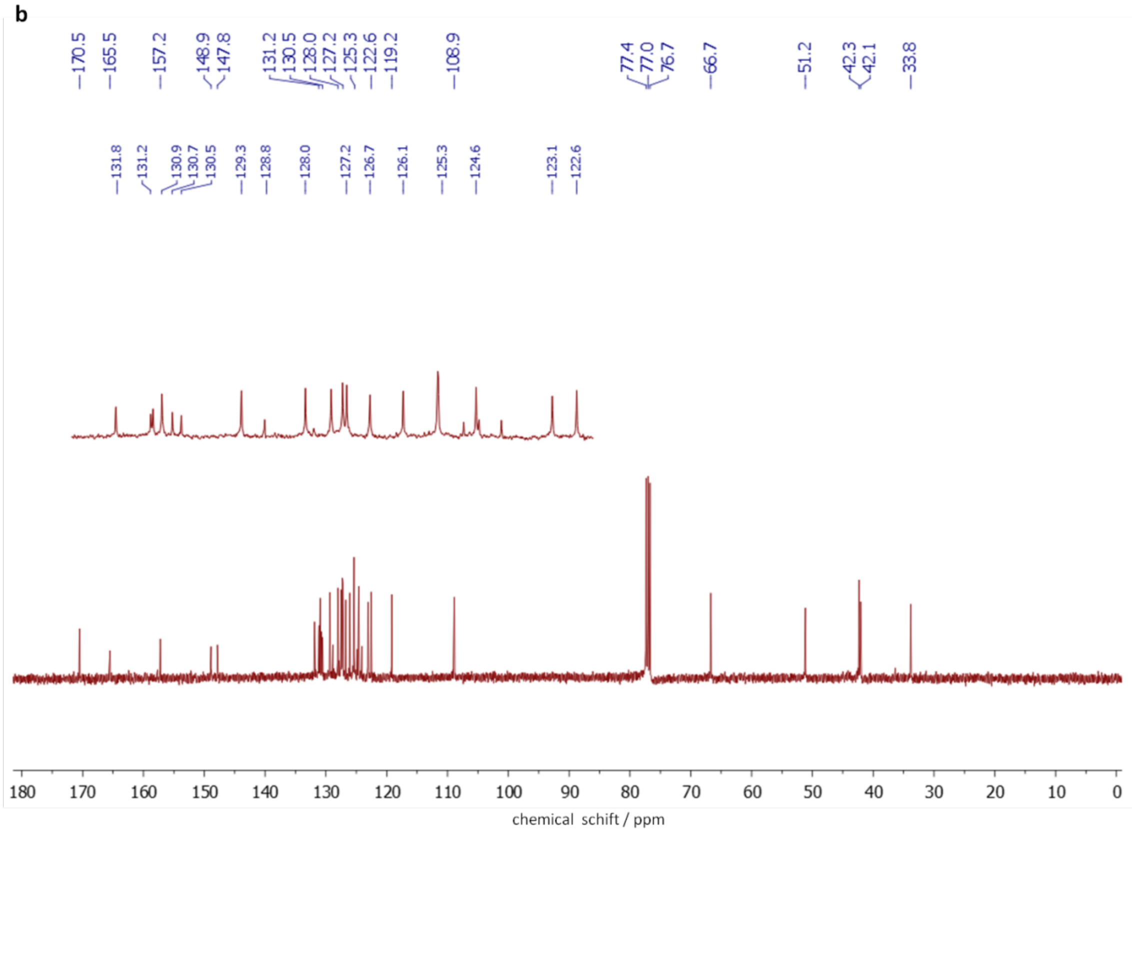

c

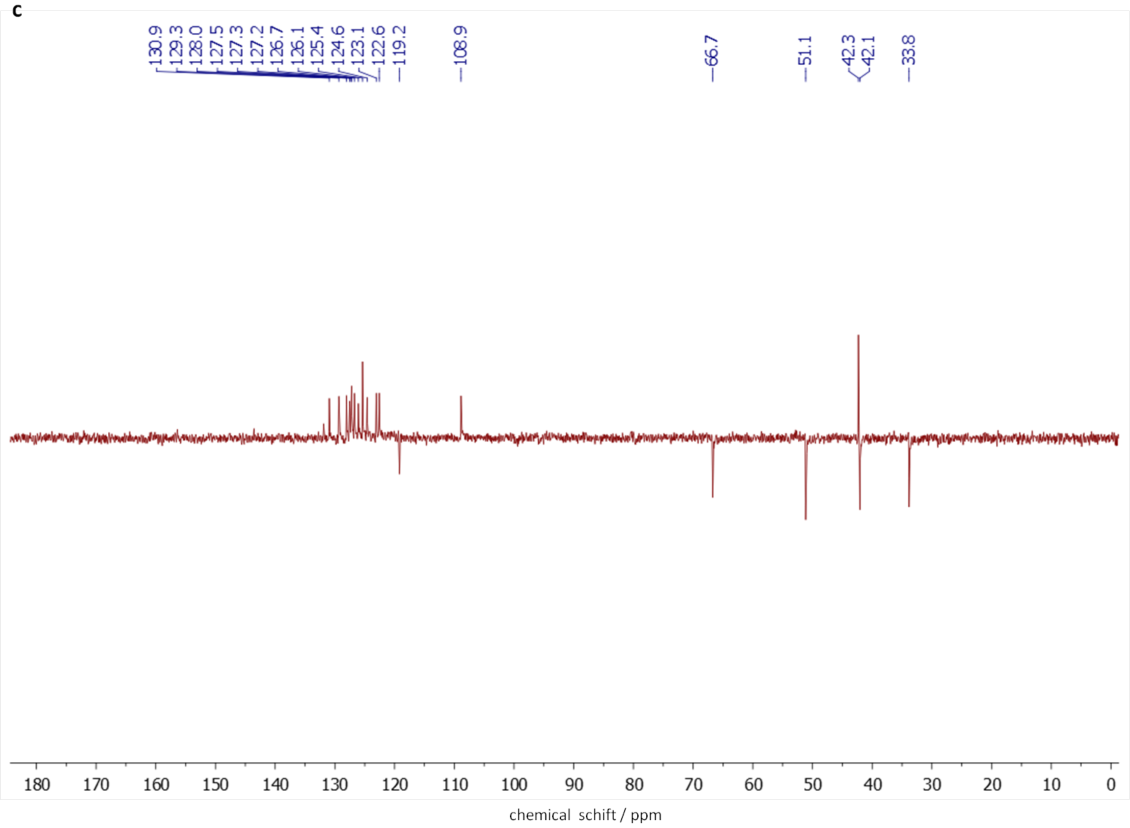

Supplementary Figure 19. **a**, ^{1}H NMR, **b**, ^{13}C NMR and **c**, DEPT of **L3**.

a

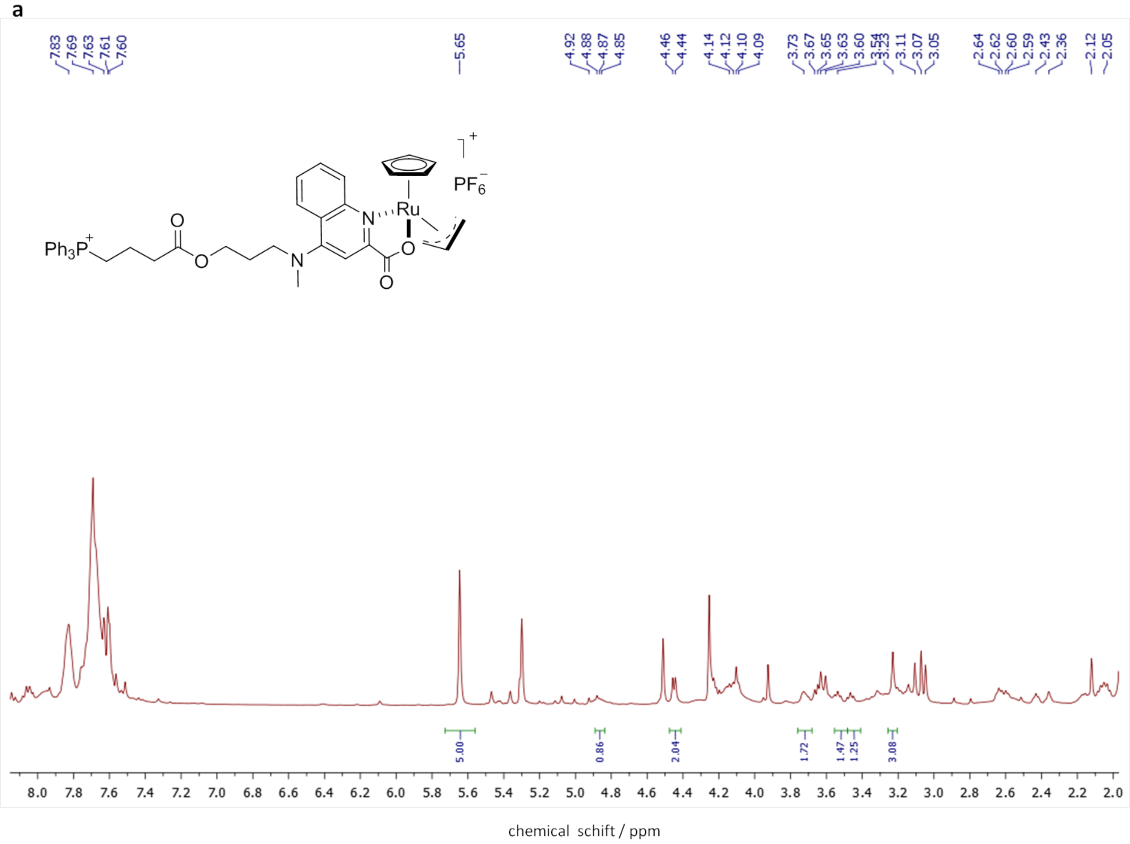

b

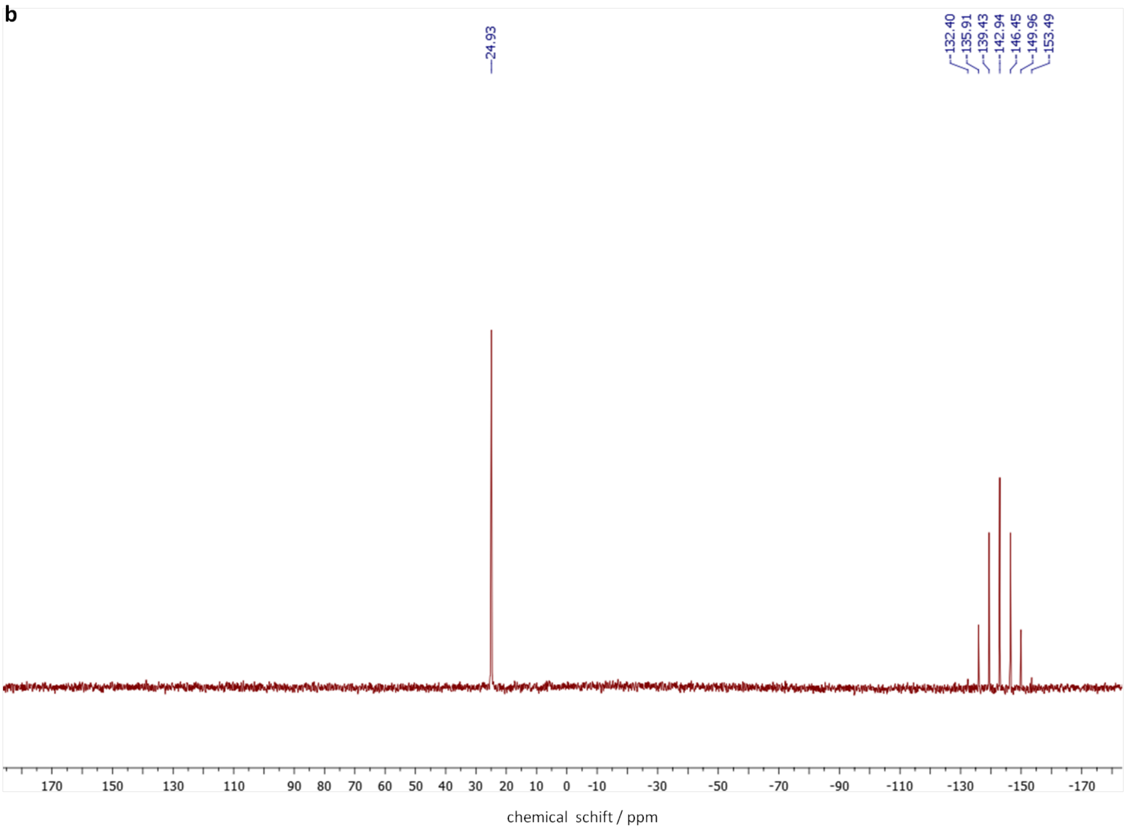

Supplementary Figure 20. **a**, ^{1}H NMR and **b**, ^{31}P NMR of **RuL1**.

a

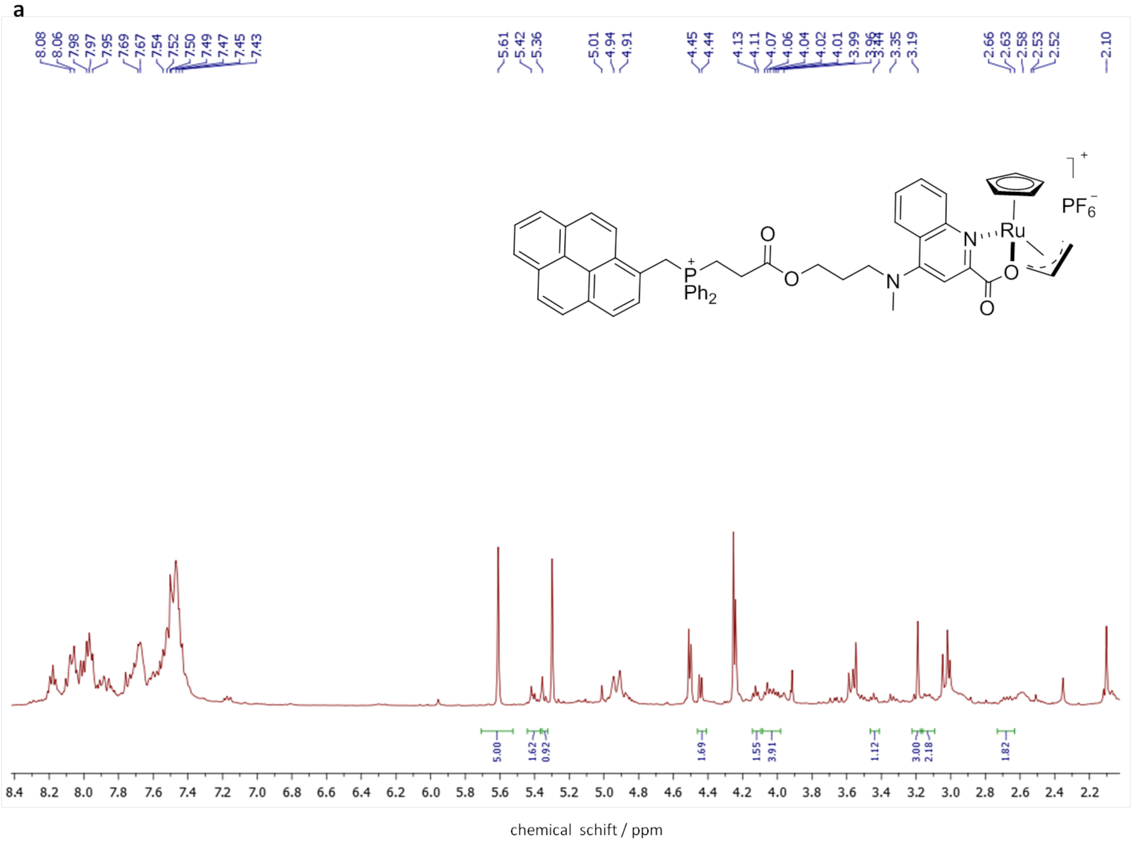

d

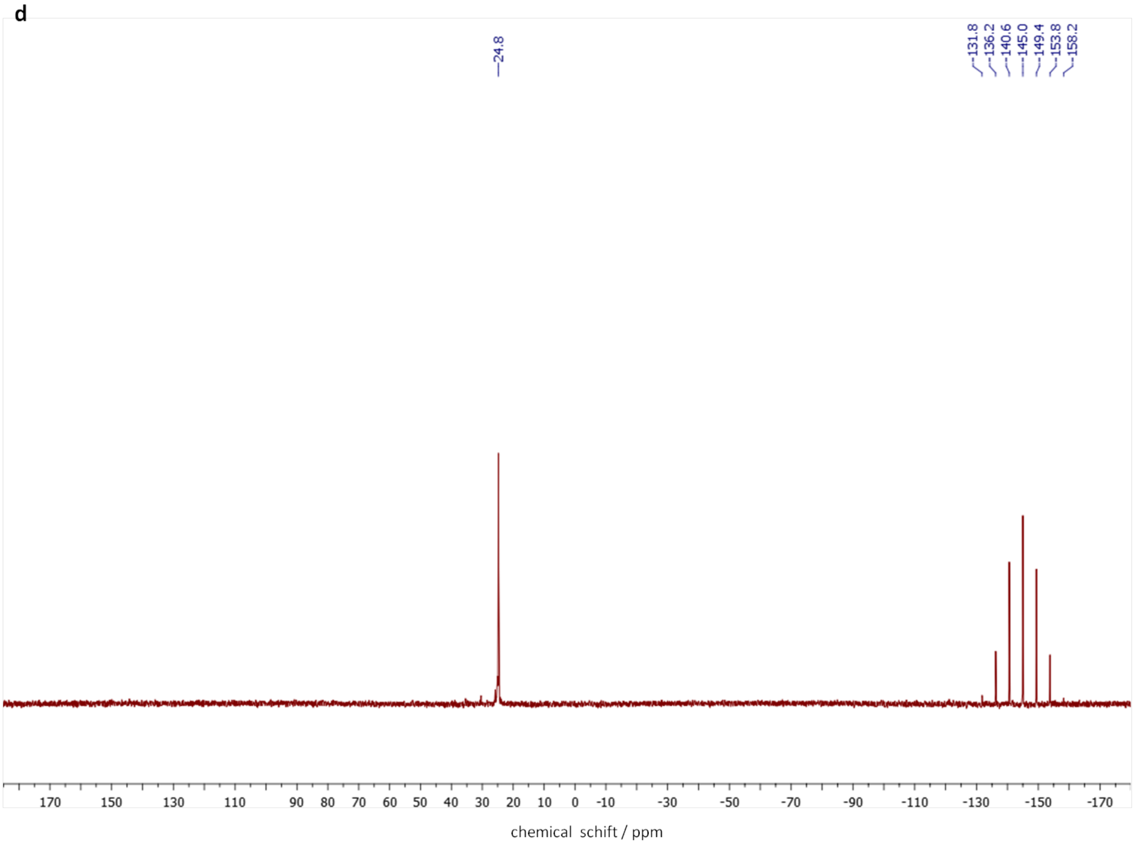

Supplementary Figure 21. **a**, ^{1}H NMR and **b**, ^{31}P NMR of **RuL2**.

a

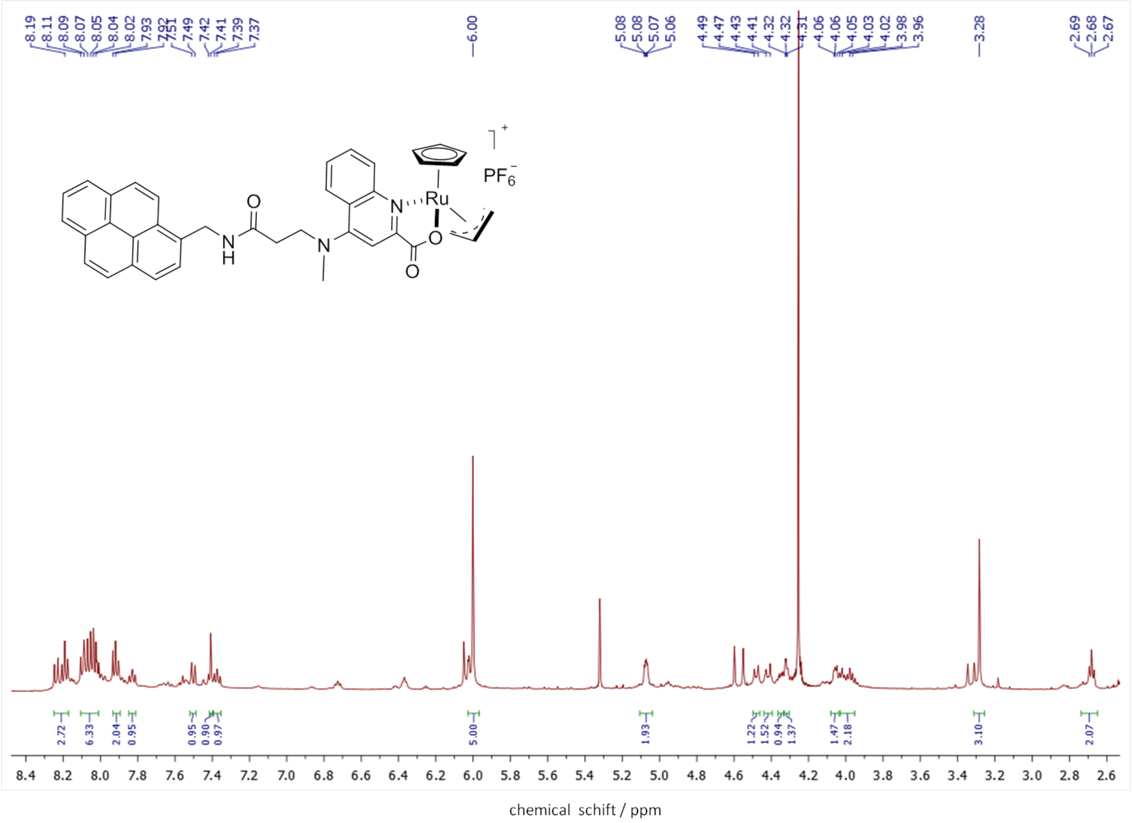

Supplementary Figure 22. **a**, ^{1}H NMR of **RuL3**.

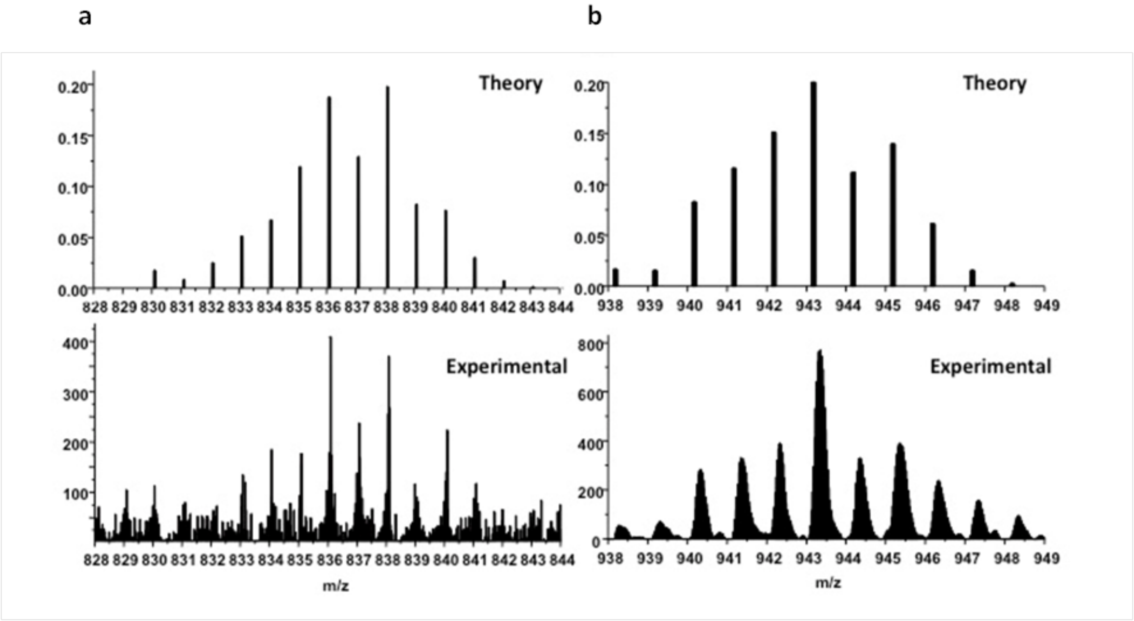

Supplementary Figure 23. Experimental and calculated **a**, HR-ESI-MS and **b**, MALDI TOF of **RuL1**.

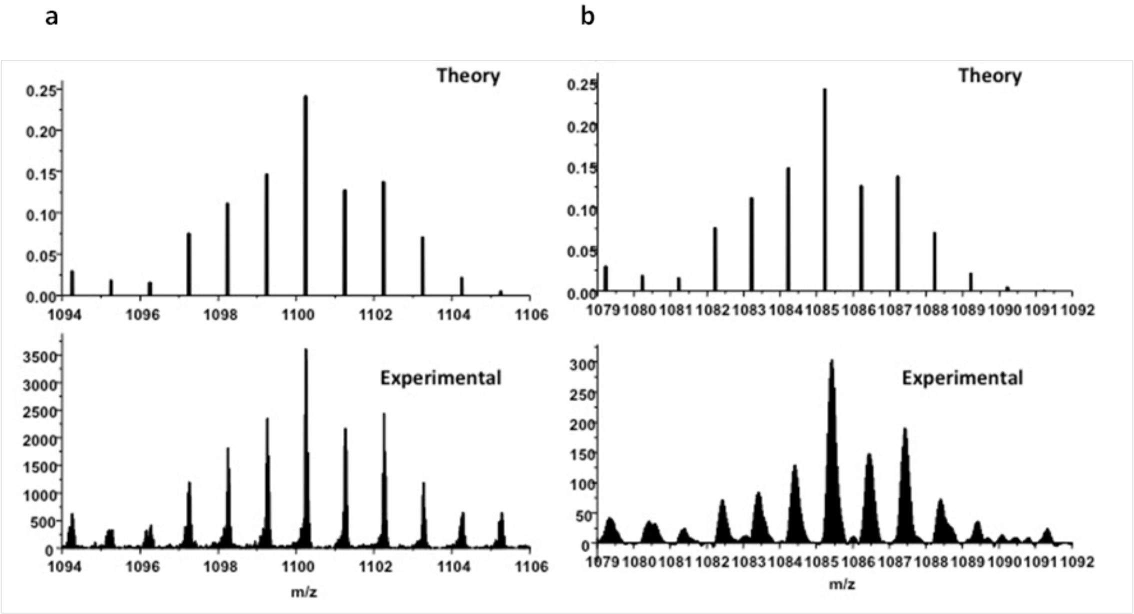

Supplementary Figure 24. Experimental and calculated **a**, HR-ESI-MS and **b**, MALDI TOF of **RuL2**.

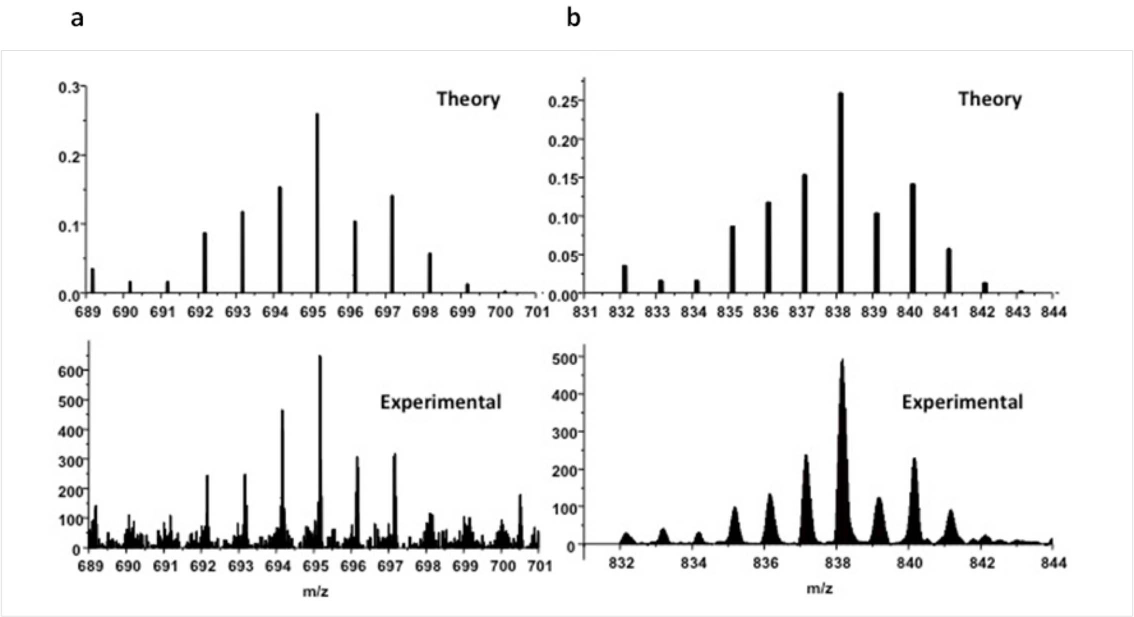

Supplementary Figure 25. Experimental and calculated HR-ESI-MS (**a**) and MALDI TOF (**b**) of **RuL3**.

Supplementary Table 1. Yields (%) in different media after 2 hours.

Catalyst	Water	PBS	Lysates
**Ru1**[4]	2	2.5	0.4
**RuL1**	6	5	5
**RuL2**	0.16	0.23	0.2
**RuL3**	14.2	10.0	12.4

Supplementary Table 2. ICP of **Ru1**, **RuL1**, **RuL2** and **RuL3** incubated in HeLa cells.

Sample	Cell Extract	[Ru] µg l^{-1}	SD	[Ru] µM	SD
**Ru1**	**Mitochondria**	21.0	0.03	0.2	0.0003
**RuL1**		**221.8**	0.01	**2.2**	0.0001
**RuL2**		**1273.0**	0.8	**12.6**	0.008
**RuL3**		51.7	0.07	0.5	0.0007
**Ru1**	**Cytosol**	19.4	0.02	0.2	0.0002
**RuL1**		77.9	0.03	0.8	0.0003
**RuL2**		81.3	0.04	0.8	0.0004
**RuL3**		47.0	0.01	0.5	0.0001

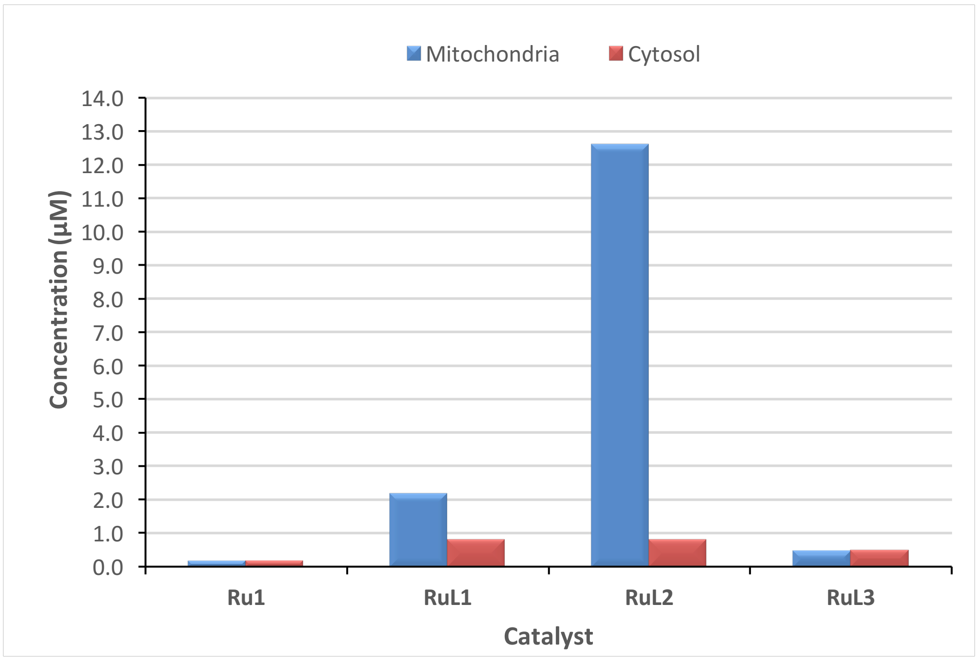

Supplementary Methods

General methods and materials for synthesis

Chemicals were purchased from *Sigma Aldrich, Fluka, ABCR, Alfa Aesar* or *Acros Organics* and used without further purification. The removal of solvents under reduced pressure was carried out on a rotary evaporator *Büchi R-210* equipped with a thermostated bath *B-491*, a vacuum regulator *V-850*, and a vacuum pump *V-700.* The solvents for organic synthesis were of reagent grade. Dry solvents were bought from *Sigma-Aldrich. N,N*-dimethylformamide and trifluoroacetic acid were purchased from *Scharlau*, dichloromethane from *Panreac* and acetonitrile from *Merck*. Water was deionized and purified on a *Millipore Milli-Q Integral* system.

Chromatographic purification of products was accomplished using flash column chromatography on *Merck* Geduran Si 60 (40 – 63 µm) silica gel (normal phase) or by reversed-phase high-performance liquid chromatography (RP-HPLC). Thin layer chromatography (TLC) was performed on *Merck* 60 (silica gel F_{254}) plates.

Products **4a** and **4b** were purified on a Büchi Sepacore preparative system consisting on a pump manager *C-615* with two pump modules *C-605* for binary solvent gradients, a fraction collector *C-660*, and UV Photometer *C-635*. Purification was made using reverse phase conditions with an isocratic regime during the first 5 min at 5% of solvent B, followed by a linear gradient from 5% to 95% of solvent B for 30 min at a flow rate of 30 mL min^{-1} (A: water with 0.1% TFA, B: methanol with 0.1% TFA) using a pre-packed preparative cartridge (150 × 40 mm) with reverse phase RP18 silica gel.

^{1}H-, $^{13}C\{^{1}H\}$ and ^{31}P NMR spectra were recorded in deuterated solvents on *Varian Mercury 300*, *Varian Inova 400* and *Bruker AMX 500* spectrometers and calibrated to the residual solvent peak, if possible. As an external reference triphenyl phosphate (-18 ppm) was used for ^{31}P NMR spectra. The chemical shifts (δ) are given in ppm, the coupling constants (J) in Hz. Multiplicities are abbreviated as follows: s = singlet, d = doublet, t = triplet, q = quartet, m = multiplet, br = broad and combinations of these. For assignment of the structures, additional 2D NMR spectra (HSQC, HMQC) were measured.

A microcentrifuge *Eppendorf 5415C* or a centrifuge *Eppendorf 5430* are used for centrifugation of the samples.

Fluorescence measurements were performed in a *Varian Cary Eclipse* Fluorescence Spectrophotometer. Fluorescence Images were obtained with an *Olympus DP-71* digital camera mounted on an *Olympus BX51* microscope or an *AndorZyla* mounted on a *Nikon TiE*.

Electrospray Ionization Mass Spectrometry (ESI/MS) was performed with a *Bruker Amazon IT/MS* using direct injection of a solution of the compound in dichloromethane into the MS. HRMS analysis were carried out in a *BRUKER AMAZON ETD*. Matrix-assisted laser desorption/ionization-time-of-flight (MALDI-TOF) mass spectra were recorded on a *Bruker Autoflex*.

4-bromoquinoline-2-carboxylic acid[1] (**1**), diallyl (3-oxo-3H-spiro[isobenzofuran-1,9'-xanthene]-3',6'-diyl)dicarbamate (**Rho-alloc**)[2] and 2,4-dinitrophenyl allyl ether (**DNP-allyl**)[3] were synthesized according to literature procedures.

1.1 Synthesis of precursors 2, 4a and 4b (Supplementary Figure 1)

Allyl 4-bromoquinoline-2-carboxylate (2)

4-Bromoquinoline-2-carboxylic acid (**1**) (200.0 mg, 0.79 mmol) was dissolved in 6 mL of DMF before $KHCO_3$ (135.0 mg, 1.35mmol) was added. To this mixture allyl bromide (0.11 mL, 1.27 mmol) was added and was left stirring at 50 °C overnight. The solvent was removed under reduced pressure and the crude obtained was extracted to DCM, washed once with NH_4Cl and three times with brine. The organic layer was dried over anhydrous $MgSO_4$, filtered and concentrated under reduced pressure. The crude was purified by flash chromatography on silica gel (AcOEt/Hex 2:1) yielding a pale yellow solid (173.0 mg, 75%).

^{1}H NMR (500 MHz, $CDCl_3$): δ (ppm) = 8.32 (s, 1H, H_{ar}), 8.17 (d, 1H, ^{3}J = 8.5 Hz, H_{ar}), 8.03 (d, 1H,^{3}J = 8.5 Hz, H_{ar}), 7.75 – 7.65 (m, 1H, H_{ar}), 7.64 – 7.54 (m, 1H, H_{ar}), 6.04 (dt, 1H,$^{3}J_{trans}$ = 23.0, $^{3}J_{cis}$ = 11.4 Hz, C*H*=CH_2), 5.40 (d, 1H,$^{3}J_{trans}$ = 18.5 Hz, CH=C*H*H), 5.26 (d, 1H, $^{3}J_{cis}$ = 10.4 Hz, CH=CH*H*), 4.90 (d, 2H,^{3}J = 5.9 Hz, CH_2).

^{13}C NMR (126 MHz, $CDCl_3$): δ (ppm) = 163.8 (CO), 147.8 (C_{ar}), 147.4 (C_{ar}), 135.0 (C_{ar}), 131.6 (*C*H=CH_2), 131.1 (CH_{ar}), 131.0 (CH_{ar}), 129.8 (CH_{ar}), 128.6 (C_{ar}), 126.5 (CH_{ar}), 124.9 (CH_{ar}), 119.4 (CH=*C*H_2), 66.9 (CH_2).

IR (neat)$\tilde{\nu}$ (cm^{-1}) = 3422, 1715, 1556, 1454, 1308, 1199, 1148, 1103, 957, 785, 753.

ESI-MS: m/z 291.95 $[M+H]^+$.

Allyl 4-[(3-hydroxypropyl)(methyl)amino]quinoline-2-carboxylate (4a)

Allyl 4-bromoquinoline-2-carboxylate (**2**) (120.0 mg, 0.41 mmol) was dissolved in 3 mL of DMSO before the temperature was raised to 100 °C. 3-*N*-methyl-propanol (**3a**) (0.2 mL, 2.05 mmol) was added dropwise. The solution was left stirring during 10 min. The reaction solution was diluted with 3 mL of water and purified by preparative RP-*Büchi Sepacore* (5-95% B in 30 min). The solvent was removed under reduced pressure yielding a yellow oil (107.0 mg, 87%).

^{1}H NMR (500 MHz, $CDCl_3$): δ (ppm) = 8.32 (d, 1H,^3J = 8.5 Hz, H_{ar}), 8.18 (d, 1H,^3J = 8.6 Hz, H_{ar}), 7.73 (t, 1H, ^3J = 7.7 Hz, H_{ar}), 7.54 (t, 1H,^3J = 7.7 Hz, H_{ar}), 7.41 (s, 1H, H_{ar}), 6.13 – 5.94 (m, 1H, C*H*=CH_2), 5.47 – 5.42 (m, 1H, CH=C*H*H), 5.35 (d, 1H,$^3J_{cis}$ = 10.3 Hz, CH=CH*H*), 4.91 (d, 2H,^3J = 6.2 Hz, CH_2), 3.97 – 3.91 (m, 2H, CH_2-OH), 3.74 (t, 2H, ^3J = 5.4 Hz, CH_2-N), 3.52 (s, 3H, CH_3), 2.65 (s, 1H, OH), 2.10 (dq, 2H,^3J = 11.6, 5.9 Hz, -CH_2-).

^{13}C NMR (126 MHz, $CDCl_3$): δ (ppm) = 160.1 (CO), 159.9 (C_{ar}), 140.7 (C_{ar}), 138.7 (C_{ar}), 133.9 (*C*H=CH_2), 130.4 (CH_{ar}), 126.5 (CH_{ar}), 126.2 (CH_{ar}), 122.1 (CH_{ar}), 120.8 (CH=CH_2), 118.5 (C_{ar}), 103.8 (CH_{ar}), 68.3 (CH_2), 58.7 (CH_2), 53.5 (CH_2), 42.6 (CH_3), 29.6 (CH_2).

IR (neat) $\tilde{\nu}$ (cm^{-1}) = 3371, 3218, 3084, 2943, 2880, 1740, 1670, 1612, 1536, 1416, 1308, 1250, 1193, 1136, 1053, 792, 715.

ESI-MS: m/z 301.13 $[M+H]^+$.

3-{{2-[(Allyloxy)carbonyl]quinolin-4-yl}(methyl)amino}propanoicacid (4b)

Allyl 4-bromoquinoline-2-carboxylate (**2**) (170.0 mg, 0.58 mmol) was dissolved in 4 mL of DMSO before the temperature was raised to 100 °C. 3-*N*-methyl-propanoic acid (**3b**) (300.0 mg, 2.91 mmol) was added. The solution was left stirring for 1 hour. The reaction solution was diluted with 4 mL of water and purified by preparative RP-*Büchi Sepacore* (5-95% B in 30 min). The solvent was removed under reduced pressure yielding a yellow oil (90.0 mg, 50%).

^{1}H-NMR (500 MHz, DMSO-d_6): δ (ppm) = 8.28 (d, 1H,^3J = 8.6 Hz, H_{ar}), 8.22 (d, 1H,^3J = 8.0 Hz, H_{ar}), 7.98 – 7.91 (m, 1H, H_{ar}), 7.68 (ddd, 1H, ^3J = 8.4, 6.9, ^4J = 1.2 Hz, H_{ar}), 7.46 (s, 1H, H_{ar}), 6.10 (ddt, 1H, $^3J_{trans}$ = 17.2, $^3J_{cis}$ = 10.5, ^3J= 5.6 Hz, C*H*=CH_2), 5.50 (dq, 1H, $^3J_{trans}$ = 17.2, $^2J_{gem}$ = 1.6 Hz, CH=C*H*H), 5.36 (dq, 1H, $^3J_{cis}$ = 10.5, $^2J_{gem}$ = 1.3 Hz, CH=CH*H*), 4.98 (dt, 2H,^3J = 5.6, ^2J = 1.4 Hz, CH_2), 3.97 (t, 2H,^3J = 5.7 Hz, CH_2-N), 2.81 (t, 2H,^3J = 7.1 Hz, CH_2), 3.40 (s, 3H, CH_3).

^{13}C-NMR (126 MHz, DMSO-d_6): δ (ppm) = 172.8 (COOH), 162.0 (CO), 159.9 (C_{ar}), 158.7 (C_{ar}), 133.4 (CH_{ar}), 132.2 (*C*H=CH_2), 126.8 (CH_{ar}), 126.7 (CH_{ar}), 126.7 (CH_{ar}), 124.1 (C_{ar}), 120.1 (C_{ar}), 119.5 (CH=*C*H_2), 105.4 (CH_{ar}), 67.5 (CH_2), 51.7 (CH_2), 42.3 (CH_3), 31.8 (CH_2).

IR (neat) $\tilde{\nu}$ (cm^{-1}) = 3339, 2928, 1740, 1676, 1610, 1539, 1421, 1203, 1135, 993, 800, 721.

ESI-MS: *m/z* 315.14 $[M+H]^+$.

1.2 Synthesis of precursor 7 (Supplementary Figure 2)

(2-Carboxyethyl)diphenyl(pyren-1-ylmethyl)phosphonium bromide (7)

1-(Bromomethyl)pyrene (**5**) (218.0 mg, 0.74 mmol) was dispersed in toluene (8 mL). To this mixture 3-(diphenylphosphino)propionic acid (**6**) (287.0 mg, 1.11 mmol) was added and the reaction mixture was left refluxing overnight. The reaction solution was hot filtered giving rise to a pale yellow solid (280.0 mg, 70%).

^{1}H NMR (500 MHz, DMSO-d$_6$): δ (ppm) = 8.31 (d, 1H,^3J = 7.6 Hz, H_{ar}), 8.26 (d, 1H, ^3J = 7.6 Hz, H_{ar}), 8.20 (t, 2H,^3J = 7.8 Hz, H_{ar}), 8.17 (d, 1H,^3J = 4.7 Hz, H_{ar}), 8.10 (d, 1H, ^3J = 9.6 Hz, H_{ar}), 8.06 (d, 1H, ^3J = 7.6 Hz, H_{ar}), 7.91 (d, 1H, ^3J = 9.3 Hz, H_{ar}), 7.79 (dd, 7H, ^3J = 12.2, 7.9 Hz, H_{ar}), 7.60 (td, 4H, ^3J = 7.7, 3.3 Hz, H_{ar}), 5.54 (d, 2H,$^2J_{HP}$ = 16.1 Hz, CH_2), 3.26 – 3.14 (m, 2H, CH_2), 2.65 – 2.51 (m, 2H, CH_2).

^{13}C NMR (126 MHz, DMSO-d$_6$): δ (ppm) = 172.2 (d, J_{CP} = 25.2 Hz, COOH), 135.3 (CH_{ar}), 134.3 (d, J_{CP} = 9.5 Hz, CH_{ar}), 131.2 (C_{ar}), 131.2 (C_{ar}), 131.0 (d, J_{CP} = 9.3 Hz, C_{ar}), 130.4 (C_{ar}), 130.2 (d, J_{CP} = 12.1 Hz, CH_{ar}), 129.7 (d, J_{CP} = 5.0 Hz, CH_{ar}), 129.2 (d, J_{CP} = 11.5 Hz, CH_{ar}), 128.4 (CH_{ar}), 127.8 (CH_{ar}), 127.7 (CH_{ar}), 127.0 (CH_{ar}), 126.1 (CH_{ar}), 125.9 (CH_{ar}), 125.3 (CH_{ar}), 124.5 (C_{ar}), 123.9 (C_{ar}), 123.4 (CH_{ar}), 122.1 (d, J_{CP} = 9.1 Hz, C_{ar}), 122.1 (C_{ar}), 117.8 (d, J_{CP} = 82.5 Hz, C_{ar}), 26.8 (CH_2), 26.1 (d, J_{CP} = 45.4 Hz, CH_2), 25.9 (CH_2), 15.7 (d, J_{CP} = 52.9 Hz, CH_2).

^{31}P NMR (202 MHz, DMSO-d6): δ (ppm) = 30.3.

IR (neat) $\tilde{\nu}$ (cm^{-1}) = 3433, 2919, 1727, 1631, 1437, 1160, 1112, 848, 744.

ESI-MS: *m/z* 473.19 $[M+H]^+$.

1.3 Synthesis of ligands L1-L3 (Supplementary Figure 3)

{4-{3-{{2-[(allyloxy)carbonyl]quinolin-4-yl}(methyl)amino}propoxy}-4-oxobutyl}triphenylphosphonium bromide (L1)

(3-Carboxypropyl)triphenylphosphonium bromide (**8**) (46.0 mg, 0.11 mmol) was dissolved in DMF (1 mL). 1-Ethyl-3-(3-dimethylaminopropyl)carbodiimide (EDC) (52.0 mg, 0.27 mmol), allyl 4-[(3-hydroxypropyl)(methyl)amino]quinoline-2-carboxylate (**4a**) (32.0 mg, 0.11 mmol) and 4-dimethylaminopyridine (26.0 mg, 0.22 mmol) were subsequently added to the solution. The reaction mixture was left stirring overnight and the solvent was removed under reduced pressure. The crude was purified by flash chromatography on silica gel (DCM/MeOH 9:1) yielding an off yellow liquid (23.0 mg, 30%).

^{1}H NMR (300 MHz, $CDCl_3$): δ (ppm) = 8.22 (dd, 2H, ^3J = 8.6, ^4J = 1.2 Hz, H_{ar}), 8.08 - 8.01 (m, 2H, H_{ar}), 7.89 – 7.80 (m, 3H, H_{ar}), 7.79 – 7.73 (m, 2H, H_{ar}), 7.71 – 7.63 (m, 7H, H_{ar}), 7.61 – 7.42 (m, 4H, H_{ar}), 6.20 – 6.04 (m, 1H, C*H*=CH_2), 5.46 (m, 1H,CH=C*H*H), 5.36 – 5.29 (m, 1H, CH=CH*H*), 4.96 (tt, 2H,$^2J_{HP}$ = 5.0, 1.3 Hz, CH_2), 4.13 – 4.04 (m, 2H, CH_2), 3.75 (t, 2H, ^3J = 6.1 Hz, CH_2), 3.55 – 3.38 (m, 4H, 2xCH_2), 3.06 (s, 3H, CH_3), 2.03 (m, 4H, 2xCH_2).

^{13}C NMR (75 MHz, $CDCl_3$): δ (ppm) = 173.1 (CO), 165.8 (CO), 158.4 (C_{ar}), 149.2 (C_{ar}), 148.1 (C_{ar}), 134.9 (CH_{ar}), 133.7 (d, J_{CP} = 10.1 Hz, CH_{ar}), 132.0 (*C*H=*C*H_2), 131.9 (CH_{ar}), 131.3 (C_{ar}), 130.4 (d, J_{CP} = 12.4 Hz, CH_{ar}), 129.7 (CH_{ar}), 129.5 (CH_{ar}), 128.5(d, J_{CP} = 12.0 Hz, CH_{ar}), 126.8 (CH_{ar}), 126.7 (CH_{ar}), 124.3 (C_{ar}), 124.0 (CH_{ar}), 119.1 (CH=*C*H_2), 118.2 (d, J_{CP} = 85.7 Hz, C_{ar}), 108.4 (CH_{ar}), 66.7 (CH_2), 62.2 (CH_2), 60.6 (CH_2), 53.0 (CH_2), 52.2 (CH_2), 41.4 (CH_3), 30.1 (CH_2), 26.5 (CH_2).

^{31}P NMR (202 MHz, $CDCl_3$): δ (ppm) = 22.8.

IR (neat) $\tilde{\nu}$ (cm^{-1}) = 3378, 3228, 3091, 2947, 1743, 1644, 1608, 1538, 1419, 1199, 1134, 798, 719.

HR-ESI-MS: *m/z* 631.2719 $[M+H]^+$, Calculated for $C_{39}H_{40}N_2O_4P$: 631.2720; found: 631.2719.

{3-{3-{{2-[(Allyloxy)carbonyl]quinolin-4-yl} (methyl)amino})propoxy}-3-oxopropyl)diphenyl(pyren-1-ylmethyl)phosphonium bromide (L2)

(2-Carboxyethyl)diphenyl(pyren-1-ylmethyl)phosphonium bromide (**7**) (41.0 mg, 0.07 mmol) was dissolved in 1 mL of DMF. 1-Ethyl-3-(3-dimethylaminopropyl)carbodiimide (EDC) (35.0 mg, 0.19 mmol), allyl 4-[(3-hydroxypropyl)(methyl)amino]quinoline-2-carboxylate (**4a**) (22.0 mg, 0.07 mmol) and 4-dimethylaminopyridine (18.0 mg, 0.15 mmol) were subsequently added to the solution. The reaction was left stirring overnight and the solvent was removed under reduced pressure. The crude was purified by flash chromatography on silica gel (DCM/MeOH 9:1) yielding a yellow oil (16.0 mg, 26%).

^{1}H NMR (500 MHz, $CDCl_3$): δ (ppm) = 8.18 (d, 1H, ^3J = 8.5 Hz, 1H_{ar}), 7.98 – 7.96 (m, 2H, H_{ar}), 7.83 (m, 3H, H_{ar}), 7.74 (m, 7H, H_{ar}), 7.65 – 7.62 (m, 1H, H_{ar}), 7.56 (m, 6H, H_{ar}), 7.48 (m, 2H, H_{ar}), 7.38 (dt, 3H, ^3J = 7.9,

4.0 Hz, H_{ar}), 6.16 – 6.08 (m, 1H, C*H*=CH_2), 5.92 (d, 2H, $^2J_{HP}$= 15.3 Hz, CH_2), 5.46 (dq, 1H, $^3J_{trans}$= 17.2, $^2J_{gem}$ = 1.4 Hz, CH=C*H*H), 5.34 – 5.31 (m, 1H, CH=CH*H*), 4.96 (dt, 2H,^3J = 5.9, 1.3 Hz, CH_2), 3.97 (t, 2H, ^3J = 6.4 Hz, CH_2), 3.66 (dt, 2H, ^3J = 12.8, 7.8 Hz, CH_2), 3.35 – 3.30 (m, 2H, CH_2), 3.02 (s, 3H, CH_3), 2.73 (dt, 2H, ^3J = 13.2, 7.7 Hz, CH_2), 1.97 – 1.91 (m, 2H, CH_2).

^{13}C-NMR (126 MHz, $CDCl_3$): δ (ppm) = 170.5 (d, J_{CP} = 14.4 Hz, CO), 165.8 (CO), 158.1 (C_{ar}), 149.2 (C_{ar}), 148.1 (C_{ar}), 134.6 (d, J_{CP} = 2.4 Hz, CH_{ar}), 134.1 (d, J_{CP} = 9.3 Hz, CH_{ar}), 132.0 (*C*H=CH_2), 131.2 (CH_{ar}), 130.7 (C_{ar}), 129.9 (C_{ar}), 129.8 (d, J_{CP} = 12.2 Hz, CH_{ar}), 129.6 (d, J_{CP} = 13.5 Hz, CH_{ar}), 129.3 (CH_{ar}), 127.6 (d, J_{CP} = 9.6 Hz,CH_{ar}), 126.7 (CH_{ar}), 126.7 (CH_{ar}), 125.8 (CH_{ar}), 125.1 (CH_{ar}), 125.0 (CH_{ar}), 124.4 (CH_{ar}), 124.2 (C_{ar}), 124.0 (CH_{ar}), 123.9 (C_{ar}), 123.6 (C_{ar}), 122.7 (CH_{ar}), 120.6 (d, J_{CP} = 10.0 Hz, C_{ar}), 119.2 (CH=*C*H_2), 116.4 (d, J_{CP} = 82.2 Hz, C_{ar}), 108.4 (CH_{ar}), 66.7 (CH_2), 63.0 (CH_2), 53.5 (CH_2), 51.9 (CH_2), 41.5 (CH_3), 27.0 (CH_2), 26.2 (CH_2), 17.3 (d, J_{CP}= 50.4 Hz,CH_2).

^{31}P NMR (202 MHz, $CDCl_3$): δ (ppm) = 25.6.

IR (neat) $\tilde{\nu}$ (cm^{-1}) = 3281, 2917, 1716, 1650, 1573, 1506, 1456, 1240, 1151, 844, 764.

HR-ESI-MS: *m/z* 755.3027 $[M]^+$; Calculated for $C_{49}H_{44}N_2O_4P$: 755.3033; found: 755.3027.

Allyl 4-{methyl{3-oxo-3-[(pyren-1-ylmethyl)amino]propyl}amino}quinoline-2-carboxylate (L3)

3-{[2-(Allyloxycarbonyl)quinolin-4-yl](methyl)amino}propanoic acid (**4b**)(18.0 mg, 0.06 mmol) was dissolved in 0.6 mL of DMF. 1-Ethyl-3-(3-dimethylaminopropyl)carbodiimide (EDC) (27.0 mg, 0.14 mmol), pyren-1-ylmethanamine hydrochloride (**9**) (15.0 mg, 0.056 mmol) and 4-dimethylaminopyridine (14.0 mg, 0.11 mmol) were subsequently added to the solution. The reaction was left stirring overnight and the solvent was removed under reduced pressure. The crude was purified by flash chromatography on silica gel (DCM/MeOH 9:1) yielding an off yellow crystalline solid (16.0 mg, 55%).

^{1}H NMR (300 MHz, $(CD_3)_2CO$): δ (ppm) = 8.34 (d, 1H, ^3J = 9.3 Hz, H_{ar}), 8.25 (t, 2H, ^3J = 7.2 Hz, H_{ar}), 8.10 – 8.07 (m, 5H, H_{ar}), 8.04 (s, 1H, H_{ar}), 8.09-7.97 (m, 2H,H_{ar}), 7.83 (brs, 1H, NH), 7.64 (t, 1H, ^3J = 8.1 Hz, H_{ar}), 7.58– 7.56 (m, 1H, H_{ar}), 7.38 (t, 1H, ^3J = 7.5 Hz, H_{ar}), 6.17-6.03 (m, 1H,C*H*=CH_2), 5.47 (d, $^3J_{trans}$ = 16.5 Hz, CH=CH*H*), 5.28 (d, 1H, $^3J_{cis}$= 10.2,CH=CH*H*), 5.08 (d, 2H, ^3J = 5.7, CH_2),4.88 (d, 2H, ^3J = 5.7 Hz, CH_2), 3.78 (t, 2H,^3J = 6.9 Hz, CH_2), 3.05 (s, 3H, CH_3), 2.71 (t, 2H,^3J = 6.9 Hz, CH_2).

^{13}C NMR (101 MHz, CDCl3): δ (ppm) = 170.5 (CONH), 165.5 (CO), 157.2 (C_{ar}), 148.9 (C_{ar}), 147.8 (C_{ar}), 131.8 (C_{ar}), 131.8 (CH_{ar}) 131.2 (C_{ar}), 131.1 (C_{ar}), 130.9 (*C*H=CH_2), 130.7 (C_{ar}), 130.5 (C_{ar}), 129.3 (CH_{ar}), 128.8 (C_{ar}), 128.0 (CH_{ar}), 127.5 (CH_{ar}), 127.3 (CH_{ar}), 127.2 (CH_{ar}), 126.7 (CH_{ar}), 126.1 (CH_{ar}), 125.3 (CH_{ar}), 125.2 (CH_{ar}), 124.9 (C_{ar}), 124.6 (CH_{ar}), 124.1 (C_{ar}), 123.1 (CH_{ar}), 122.6 (CH_{ar}), 119.2 (CH=*C*H_2), 108.9 (CH_{ar}), 66.7 (CH_2), 51.1 (CH_2), 42.3 (CH_3), 42.1 (CH_2), 33.8 (CH_2).

IR (neat) $\tilde{\nu}$ (cm^{-1}) = 3403, 2926, 2861, 11730, 1619, 1575, 1436, 1356, 1238, 1113, 1027, 850, 739.

HR-ESI-MS: *m/z* 528.2284 $[M+H]^+$; Calculated for $C_{34}H_{30}N_3O_3$: 528.2282; found: 528.2284.

1.4 General procedure for the synthesis of the catalysts RuL1-RuL3 (Supplementary Figure 4)

Synthesis of the Ruthenium catalysts RuL1-RuL3

To a 10 mM solution of **L1-L3** (0.02 mmol) in anhydrous DCM (0.17 mL) was added 1 eq of $[RuCp(CH_3CN)_3]PF_6$ (0.02 mmol). The reaction mixture was left stirring overnight at room temperature. The reaction mixture was centrifuged, the supernatant was collected and the solvent was removed under reduced pressure yielding a dark solid. Assignation of the ^{1}H NMR of complexes **RuL1** and **RuL2** was not possible due to problems of aggregation of these compounds. Nevertheless, these spectra are shown in Supplementary Fig. 16 and Fig. 17.

RuL1 (12.0 mg, 65%)

^{1}H NMR (500 MHz, (CD_2Cl_2)

^{31}P NMR (202 MHz, DMSO-d_6): δ (ppm) = 24.9, -142.9 (septet, $^1J_{PF}$ = 709.0 Hz, PF_6^-).

IR (neat) $\tilde{\nu}$ (cm^{-1}) = 3436, 2925, 1730, 1648, 1585, 1540, 1439, 1412, 1114, 843, 724.

HR-ESI-MS: m/z 836.0953 $[RuCp(L1)Br]^+$ Calculated for $C_{41}H_{40}BrN_2O_4PRu^+$: 836.0953; found: 836.0957. MALDI-TOF: m/z 943.1808 $[RuCp(L1)(Allyl)Br]^+$ Calculated for $C_{44}H_{45}F_6N_2O_4P_2Ru^+$: 943.1808; found: 943.298.

RuL2 (13 mg, 68%)

^{1}H NMR (400 MHz, (CD_2Cl_2)

^{31}P NMR (202 MHz, CD_2Cl_2): δ (ppm) = 24.8, -145.0 (septet, $^1J_{PF}$= 709.0 Hz, PF_6^-).

IR (neat) $\tilde{\nu}$ (cm^{-1}) = 3437, 2924, 1734, 1643, 1585, 1438, 1411, 1113, 844, 741.

HR-ESI-MS: m/z 1100.2461 $[RuCp(L2)(Allyl)(PF_6)](CH_3OH)^+$ Calculated for $C_{55}H_{54}F_6N_2O_5P_2Ru$: 1100.2461; found: 1100.2574. MALDI-TOF: m/z 943.1808 $[RuCp(L2)(Allyl)PF_6](H_2O)^+$ Calculated for $C_{54}H_{51}F_6N_2O_5P_2Ru$: 1085.223; found: 1085.430.

RuL3 (10 mg, 63%)

^{1}H NMR (400 MHz, (CD_2Cl_2): 8.25-7.36 (several multiplets, 14 H, H_{ar}), 6.00 (s, 5H, C_5H_5), 5.07 (br s, CH_2), 4.49-4.47 (m, 1H, $H_{allylic}$), 4.43-4.40 (m, 1H, $H_{allylic}$), 4.36-4.30 (m, 2H, $H_{allylic}$), 4.07-4.05 (m, 1H, $H_{allylic}$), 4.03-3.95 (m, 2H, CH_2), 3.28 (s, 3H, CH_3), 2.68 (t, 2H, ^3J = 6.5 Hz, CH_2).

IR (neat) $\tilde{\nu}$ (cm^{-1}) = 3264, 3049, 2922, 1649, 1583, 1541, 1411, 1353, 1269, 1187, 1101, 1029, 846, 765.

HR-ESI-MS: m/z 695.1727 {[RuCp(L3)(Allyl)]+H}$^+$ Calculated for $C_{39}H_{34}N_3O_3Ru$: 695.1727; found: 695.1927. MALDI-TOF: m/z 838.121 [RuCp(L3)(Allyl)(PF$_6$)]$^+$ Calculated for $C_{39}H_{33}F_6N_3O_3PRu$: 838.121; found: 838.183.

2. Catalysis experiments in vitro

The catalytic deprotection of **Rho-alloc** to **Rho** under biologically relevant conditions was performed in a 0.5 mL Eppendorf tube. For this purpose, fresh solutions of **Rho-alloc** (6 µL, 10 mM in DMSO, 1.0 eq) and glutathione (6 µL, 100 mM in water, 10 eq) were either added to water or PBS (107.7 µL). In the experiments performed in HeLa cell lysates, glutathione was not needed and the fresh solution of **Rho-alloc** was added to 113.7 µL of the lysate suspension. After mixing, a solution of the catalyst (0.3 µL, 10 mM in DMSO, 0.05 eq) was added. The reaction mixture was mixed again and incubated for 4 h at 37 °C. At regular intervals, aliquots of the reaction (2 µL) were diluted with PBS (998 µL) in a quartz Hellma® fluorescence cuvette with a pathlength 10x4 mm, chamber volume 1.4 mL to reduce the substrate concentration to 1 µM and then analyzed in a *Varian Cary Eclipse* Fluorescence Spectrophotometer. The samples were excited at 490 nm and the emission spectra were recorded in the interval 495-700 nm. The yields were calculated by the ratio between the fluorescence of the uncaged product and the measured emission of a solution 1 µM of Rhodamine 110 (**Rho**). Every value is the average value of three measurements (Supplementary Table 1).

Preparation of cell lysates: approximately $3x10^6$growing HeLa cells were washed with PBS, scraped with a rubber policeman into 0.5 mL of PBS and sonicated intensely for 2 rounds of 30 seconds each with a 30 seconds cooling period in between. The protein concentration of the lysates was quantified by DC™ Protein Assay (BioRad) and equalised to 1 mg mL^{-1} for reproducibility among experiments.

3. Spectroscopy studies

For the spectroscopic studies of the ruthenium catalyst a fresh solution of the metal complexes (10 mM in DMSO) was prepared and diluted into a quartz Hellma® fluorescence cuvette with a pathlength 10x10 mm, chamber volume 3.5 mL to reduce the catalyst concentration to 3 µM. The samples were analysed in a *Jasco V-630*UV-Vis spectrophotometer and in a *Varian Cary Eclipse* Fluorescence Spectrophotometer. Samples were recorded at 25 °C. UV-Vis analyses were performed in the interval of 200-700 nm. For the fluorescence studies, **RuL2** solution was excited at 350 nm and the emission spectrum was recorded in the interval 355-700 nm (Supplementary Figure 8a). In the case of the **RuL3** solution λ_{exc} was 345 and the spectrum was performed between 350 and 700 nm (Supplementary Figure 8b). The excitation fluorescence spectra of **RuL2** and **RuL3** were recorded in the interval 200-360 nm with λ_{em} 380 and 375 nm respectively.

4. Experiments with cells

All cell lines were cultured in DMEM (Dulbecco's modified Eagle's medium) supplemented with 10%

(v/v) fetal bovine serum (FBS), 5 mM glutamine, penicillin (100 units mL^{-1}) and streptomycin (100 units mL^{-1}) (all from *Invitrogen*). Proliferating cell cultures were maintained in a 5% CO_2 humidified incubator at 37 °C. Unless otherwise specified, all incubations were performed in DMEM supplemented with 5% of FBS (DMEM-FBS) at 37 °C.

4.1 Viability Assays

The toxicity of the catalysts **Ru1**, **RuL1**-**RuL3** was tested by MTT assay in HeLa, Vero and A549 cell lines as follows: 150000 cells per well were seeded in 96 well plates one day before treatment with different concentrations of the catalysts. After 24 h of incubation, Thiazolyl Blue Tetrazolium Bromide (*Sigma*) was added to the cell culture medium to a final concentration of 0.5 mg mL^{-1}. Cells were then incubated for 4 h to allow the formation of formazan precipitates by metabolically active cells. A detergent solution of 10% SDS (sodium dodecyl sulfate) and 0.01 M HCl was then added and the plate was incubated overnight at room temperature to allow the solubilisation of the precipitates. The quantity of formazan in each well (directly proportional to the number of viable cells) was measured by recording changes in absorbance at 570 nm in a microtiter plate reading spectrophotometer (*Tecan Infinite 200 PRO*) (Supplementary Figure 5).

4.2 Catalysis experiments in living cells

4.2.1 Uncaging of Rho-alloc

- Cellular retention of the catalysts:

Rho-alloc preincubation: Cells growing on glass coverslips were incubated in DMEM-FBS containing **Rho-alloc** (100 µM) for 30 min. A washing step in PBS for 20 min was performed followed by an incubation in DMEM-FBS containing the corresponding catalyst (25 µM) for 15 minutes. Finally, the cells were washed for 4 min in PBS before observation in the fluorescence microscope.

Catalyst preincubation: Cells growing on glass coverslips were incubated with one of the different metal catalysts (25 µM) for 15 minutes in DMEM-FBS. Then the cells were washed for 20 min in PBS and incubated in 100 µM **Rho-alloc** in DMEM-FBS for 30 min. The coverslips were then washed for 4 min in PBS and observed in the fluorescence microscope.

Digital pictures of the different samples were taken under identical conditions of gain and exposure.

- Intracellular localization of the catalysis

Cells growing on glass coverslips were incubated with either catalyst **Ru1**, **RuL1**, **RuL2** or **RuL3** (50 µM) and TMRE (100 nM) for 30 minutes. Cells were then washed twice with DMEM-FBS and incubated with **Rho-alloc** (100 µM) for 30 minutes. Prior to observation by fluorescence microscopy, the samples were washed twice with fresh DMEM-FBS. The coverslips were observed *in vivo* in a fluorescence microscope

equipped with adequate filters. Digital pictures of the different samples were taken under identical conditions of gain and exposure (Supplementary FIgures 6 and 7).

- Calculation of Mander's coefficients

The calculation was performed on dual-colour images from fluorescent microscopy experiments as the one represented in Fig. 4. These coefficients were calculated with the public domain tool JACoP (http://rsb.info.nih.gov/ij/ plugins/track/jacop.html) implemented in the program *ImageJ*.[5]

Manders' overlap coefficient (MOC) is based on the Pearson's correlation coefficient but it doesn't take into account the average intensity values in its mathematical expression (Manders *et al.*, 1993).[6] As a result, this parameter is almost independent of signal proportionality and is instead only sensitive to co-occurrence. Mander's coefficient varies from 0 to 1, the former corresponding to non-overlapping images and the latter reflecting 100% colocalization between both images. Since Mander's coefficient is very sensitive to noise, a threshold to the estimated value of background, equalized for every image, was used as zero.

4.2.2 Catalysis in conditions of mitochondrial membrane depolarization

Cells growing on glass coverslips were incubated with either catalyst **Ru1**, **RuL1-RuL3** (50 µM) and MitoTracker red (200 nM) for 30 minutes. Medium was removed and the cells were washed twice with DMEM-FBS. Afterwards, cells were incubated with FCCP (100 µM) for 10 minutes. When the catalysis was also studied, after incubation with FCCP, **Rho-alloc** (100 µM) was added to the incubation medium for 30 minutes. The coverslips were then observed *in vivo* in a fluorescence microscope equipped with adequate filters. Digital pictures of the different samples were taken under identical conditions of gain and exposure (Supplementary Figure 10).

4.2.3 Uncaging of DNP-allyl

Cells growing on glass coverslips were incubated with either catalyst **RuL2** or **RuL3** (50 µM) for 30 minutes or left untreated. Cells were then washed twice with DMEM-FBS and incubated for 30 min with the caged **DNP-allyl** (150 µM) or uncaged **DNP** (500 µM) as a positive control. Finally, TMRE (100 nM) was added to the incubation medium for 10 minutes. The coverslips were then observed *in vivo* in a fluorescence microscope equipped with adequate filters. Digital pictures of the different samples were taken under identical conditions of gain and exposure (Supplementary Figures 11 and 12).

Microscopy settings

Images were obtained with an *Olympus DP-71* digital camera mounted on an *Olympus BX51* microscope or an *AndorZyla* mounted on a *Nikon TiE*. Images were further processed with Image J or NIS software (Nikon).

The parameters of the fluorescent channels were the following:

For the Olympus BX51: filter cube U-MWU2: excitation filter (BP) 330-385 nm, emission filter (LP) 420 nm and dichromatic mirror (DM) 400 nm; filter cube U-MWB2: BP 460-490 nm, LP 520 nm and DM 500 nm; filter cube UMNG2: BP 530-550 nm, LP 590 nm and DM 570 nm.

For the Nikon (Semrock): filter cube DAPI-1160B-000: BP 387/11 nm, LP 447/60 nm and DM 409 nm; filter cube FITC-3540C-000: BP 482/35 nm, LP 536/40 nm and DM 506 nm; filter cube TRITC-B-000: BP 543/22 nm, LP 593/40 nm and DM 562 nm.

ICP analysis

For the ICP measurements a total of $3x10^6$ HeLa cells growing in 6 well plates were treated with 50 µM of the different catalysts in DMEM-FBS for 60 minutes. Then, a commercial kit (Mitochondria Isolation Kit -Thermo-Fisher Scientific-) was used to isolate mitochondrial and cytosolic cellular fractions. The obtained fractions were digested in duplicate in HNO_3/H_2O_2 by microwave heating and analyzed (Supplementary Table 2).

Supplementary References

1. Kato, Y., Okada, S., Tomimoto, K. & Mase, T. A facile bromination of hydroxyheteroarenes. *Tetrahedron Lett.***42,** 4849–4851 (2001).

2. Streu, C. & Meggers, E. Ruthenium-induced allylcarbamate cleavage in living cells. *Angew. Chem. Int. Ed.* **45,** 5645–5648 (2006).

3. Zhou, Meiyun; Li, Yiqun; Kuang, Jinyong; Xu, Xinming; Huang, G. Novel [bmim]PF6/H2O bi-phase system for the Williamson reactions. *Huaxue Tongbao***67,** w66/1–w66/3 (2004).

4. Volker, T., Dempwolff, F., Graumann, P. L. & Meggers, E. Progress towards bioorthogonal catalysis with organometallic compounds. *Angew. Chem. Int. Ed.***53,** 10536–10540 (2014).

5. Bolte, S. & Cordelières, F. P. A guided tour into subcellular colocalization analysis in light microscopy. *J. Microsc.***224,** 213–232 (2006).

6. Manders, E. M. M., Verbeek, F. J. & Ate, J. A. Measurement of co-localisation of objects in dual-colour confocal images. *J. Microsc.***169,** 375–382 (1993).
